# Prenatal Exposure to Neonicotinoid Insecticides and Neurological and Cognitive Development in Preschool Children: Evidence from a Birth Cohort in Guangxi, China

**DOI:** 10.3390/toxics14050445

**Published:** 2026-05-20

**Authors:** Qingqing Liang, Haiyan Li, Lihong Zhou, Changhui Mu, Mengrui Lin, Qian Liao, Shun Liu, Xiaoqiang Qiu, Dongping Huang, Dongxiang Pan, Xiaoyun Zeng

**Affiliations:** 1 Department of Epidemiology and Health Statistics, School of Public Health, Guangxi Medical University, Nanning 530021, China; liangqingqing@sr.gxmu.edu.cn (Q.L.); lhy@sr.gxmu.edu.cn (H.L.); 18277103236@163.com (L.Z.); maaiya@163.com (Q.L.); xqqiu9999@163.com (X.Q.); 2 Department of Sanitary Inspection, School of Public Health, Guangxi Medical University, Nanning 530021, China; everyday202605@163.com (C.M.); linmengrui1996@163.com (M.L.); dongpinghuang@gxmu.edu.cn (D.H.); 3 Department of Maternal, Child and Adolescent Health, School of Public Health, Guangxi Medical University, Nanning 530021, China; liushun@gxmu.edu.cn; 4 China (Guangxi)-ASEAN Engineering Research Center of Big Data for Public Health, Guangxi Medical University, Nanning 530021, China; 5 Department of Epidemiology and Health Statistics, School of Public Health, Guilin Medical University, Guilin 541199, China

**Keywords:** neonicotinoid insecticides, prenatal exposure, neurocognitive development, WPPSI-IV, ASQ, preschool children

## Abstract

Neonicotinoid insecticides (NEOs) are widely used globally, leading to human exposure including pregnant women, and may pose risks of neurocognitive toxicity. In this study, we analyzed 114 mother–child pairs from the Guangxi Zhuang birth cohort. Umbilical cord plasma concentrations of 10 NEOs were measured using ultra-high-performance liquid chromatography–mass spectrometry (UPLC–MS), and child neurocognitive development was assessed using the Wechsler Preschool and Primary Scale of Intelligence, Fourth Edition (WPPSI-IV) and the Ages and Stages Questionnaire, Third Edition (ASQ-3). NEOs were frequently detected, with detection rates ranging from 15.8% to 96.5%, and dinotefuran (DIN) showed the highest prevalence. Prenatal exposure to several NEOs was associated with lower neurocognitive scores. Specifically, DIN and clothianidin (CLO) exposure were associated with lower Full-Scale Intelligence Quotient (FSIQ), while thiacloprid (THIA) exposure was linked to poorer communication performance. In addition, imidacloprid (IMI) and THIA exposure were associated with reduced gross motor function, and thiamethoxam (TMX) was further associated with reduced fine motor development. Mixed exposure analysis suggested a negative but non-significant association between overall NEO exposure and FSIQ or fine motor outcomes. These findings suggest a potential association between prenatal exposure to NEOs and neurocognitive development in preschool children, highlighting the need for further research to inform public health strategies.

## 1. Introduction

Neurodevelopmental disorders (NDDs) are conditions that emerge early in life and are characterized by impairments in brain development, cognition, and behavior, leading to lifelong challenges for affected individuals and their families [1]. Global burden of disease studies indicate that NDDs represent a major and growing public health challenge for children [2,3,4,5]. This not only severely restricts children’s development potential but also imposes a huge economic and care burden on families and society [6]. Among the various factors influencing neurocognitive development, exposure to environmental toxicants is a critical yet insufficiently understood area [7,8,9,10,11,12,13].

Neonicotinoids insecticides (NEOs) are widely used insecticides that act on nicotinic acetylcholine receptors and are valued for their high selectivity, bioavailability, and relative environmental stability [14,15]. China is the world’s largest producer and consumer of NEOs, with imidacloprid production exceeding half of global output as early as 2012 [16,17]. Their extensive use has resulted in widespread environmental contamination [18], with NEOs detected in water [19], soil [20,21,22], air [23], and food [20,24], leading to multiple routes of human exposure. Biomonitoring studies in China have detected multiple NEOs and their metabolites in urine samples from the general population, with high detection rates and measurable concentrations [25,26]. NEOs and their metabolites have also been identified in human cerebrospinal fluid [27], breast milk [28,29], and serum [30]. What is more crucial is that NEOs can also be detected in the bodies of pregnant women and newborns [29,31]. A report indicates that the median daily intake of Acetamiprid, Clothianidin and Thiamethoxam by Chinese pregnant women was approximately 64.0, 3.46 and 3.58 nanograms per kilogram body weight per day, respectively [32]. These findings suggest that the pregnant women had been exposed to NEOs on a widespread basis.

NEOs effects by selectively activating nicotinic acetylcholine receptors (nAChRs) in insect nervous system [33]. In vertebrates, including humans, nAChRs are widely expressed in the central nervous system and play crucial roles in cognitive processes such as learning, memory, attention, and synaptic plasticity [34,35]. Notably, nAChRs do not function independently but form functional signaling networks with other neurotransmitter receptors, including glutamate receptors (GluRs) and N-methyl-D-aspartate receptors (NMDARs) [34,36]. Dysregulation of nAChR signaling has been implicated in a range of neurodegenerative and neuropsychiatric disorders, underscoring its importance in brain function [37,38]. Importantly, human and insect nAChR subunits share substantial structural and sequence homology within key functional domains, suggesting that neonicotinoids may interact with mammalian nAChRs [39,40]. In addition, experimental evidence indicates that certain neonicotinoids can cross the blood–brain barrier (BBB) and reach brain tissue in animal models [41,42]. Although pre-market experiments indicated that NEOs have very low acute toxicity to adult mammals, these findings do not preclude the possibility that maternal exposure could affect fetal neural development, given that NEOs can cross the placenta and BBB [43,44].

Moreover, the existing epidemiological evidence supports the possibility that NEOs induce neurodevelopmental toxicity. A cohort study in Taiwan found that during childhood, especially for boys, exposure to NEOs has a negative impact on neurodevelopment [45]. The levels of urinary NEOs metabolites are associated with neurological symptoms in both children and adults, including memory loss and autism spectrum disorders [46,47]. Gunier et al. found that prenatal exposure to NEOs was associated with lower child IQ using a geospatial analytical assessment of maternal housing adjacent to agricultural application sites [48]. Studies on the use of NEOs in prenatal families have shown that exposure to NEOs is associated with an increased risk of autism in children [49]. The use of NEOs during pregnancy increases the risk of neural tube defects [50], autism spectrum disorders [51] and lower intelligence quotient [48] in 7-year-old children. However, research on prenatal pesticide exposure and children’s neurocognitive development is still far from conclusive, as some studies have reported an insignificant association between pesticide exposure and cognitive development [52,53,54]. Additionally, other research has shown that there is no statistically significant correlation between the concentrations of urinary NEOs (dm-ACE, CLO, DIN, and THX) in pregnant women during pregnancy and the neurodevelopmental scores of children under the age of 4 [55]. A study from Taiwan concluded that exposure to NEOs during the late stage of pregnancy does not affect neural development [45].

However, most epidemiological studies focus on postnatal or childhood exposure, while evidence for the most vulnerable fetal period (i.e., pre-natal exposure) is relatively weak and inconsistent. Most existing studies assess human pesticide exposure through urine samples, which may be less capable of accurately reflecting the pesticide exposure levels of the mother compared to a blood sample [56]. Therefore, to fill this knowledge gap, this study explored the association between single and mixed prenatal exposure to NEOs in umbilical cord blood and neurocognitive development in preschool children based on the Guangxi Zhuang birth cohort in China.

## 2. Materials and Methods

### 2.1. Study Population

This study utilized the data collected in Guangxi Zhuang Birth Cohort (GZBC), which is a multi-purpose cohort study conducted in the maternal and child health hospitals and people’s hospitals of various counties in Guangxi Zhuang Autonomous Region, China. The detailed information of GZBC has been described in previous studies [57]. The inclusion criteria of this study: (a) natural conception; (b) planned to receive prenatal care and delivery in the research hospital with barrier-free communication. Subjects will be excluded if they have any of the following conditions: (a) Lack of fetal umbilical cord plasma samples; (b) Lack of data of neurodevelopmental assessment.

A total of 7 mother–child pairs without available blood samples and 18 pairs with incomplete information due to loss to follow-up or missing data were excluded. Ultimately, 114 mother–child pairs were included in the analysis.

After obtaining written informed consent from the mothers, structured questionnaires were administered to collect information on parental demographics, socioeconomic status, educational attainment, diet, and lifestyle factors. This study was conducted in accordance with the Declaration of Helsinki and approved by the Ethics Review Committee of Guangxi Medical University (approval number: 20140305-001, date of approval: 5 March 2014). Written informed consent was obtained from all participants.

### 2.2. NEOs Exposure Assessment

Blood sample collection is carried out by professional medical staff using anticoagulant blood collection containers (BD Biosciences, Franklin Lakes, NJ, USA) to draw 5 mL of umbilical cord blood after the fetus is delivered. The blood samples were centrifuged for 10 min at 4 °C and 4000 r/min by a low-temperature, high-speed centrifuge (Model L535R, Hunan Cence Instrument Co., Ltd., Changsha, China). The upper plasma layer was taken and stored in an ultra-low temperature freezer (Model DW-86L338J, Qingdao Haier Biomedical Co., Ltd., Qingdao, China) at −80 °C for further measurement of NEOs.

A total of nine NEOs, including acetamiprid (ACE), dinotefuran (DIN), clothianidin (CLO), flupyradifurone (FLU), sulfoxaflor (SUL), thiacloprid (THIA), imidacloprid (IMI), nitenpyram (NIT), and thiamethoxam (TMX), together with one metabolite, acetamiprid-N-desmethyl (NACE), were analyzed in umbilical cord plasma samples. Analytical standards were obtained from Dr. Ehrenstorfer GmbH (Augsburg, Germany) (catalog numbers: ACE, DRE-C10013000; DIN, DRE-C12820000; CLO, DRE-C11691700; FLU, DRE-C13802300; SUL, DRE-C17015000; THIA, DRE-C17451000; IMI, DRE-C14283700; NIT, DRE-C15535000; TMX, DRE-C17453000; NACE, DRE-C10013200). All standards were of high purity (≥98%) and stored at −20 °C according to the manufacturer’s instructions. Sample preparation was performed using a liquid–liquid extraction method, and extracts were concentrated under vacuum centrifugation (Jiaimu Technology Co., Ltd., Suzhou, China). Target analytes were quantified using ultra-high-performance liquid chromatography-mass spectrometry (UPLC-MS, Waters Corp., Milford, MA, USA). Representative chromatograms of the NEOs standards are provided in the Supplementary Information (Figure S1). Detailed measurement schemes have been described in previous studies [58]. Briefly, calibration curves were established over a concentration range of 0.05–50 ng/mL, with coefficients of determination (R^2^) ≥ 0.990. Procedural blanks were included in each analytical batch to ensure data quality. Mean recoveries ranged from 80.0% to 125.0%, and intra- and inter-day relative standard deviations (RSDs) were below 9.0%, indicating good analytical precision. The limits of detection (LODs) for the 10 NEOs ranged from 0.025 to 0.05 ng/mL. Concentrations below the LOD were imputed as LOD divided by √2.

### 2.3. Neurological and Cognitive Development Assessment

This study initiated follow-up of children aged 3 to 6 years and their families in 2021. Children’s cognitive abilities were assessed using the Chinese version of the Wechsler Preschool and Primary Scale of Intelligence-Fourth Edition (WPPSI-IV) [59], based on a standardized classification system. Cognitive function was evaluated across five core domains, including the Verbal Comprehension Index (VCI), Visual Spatial Index (VSI), Fluid Reasoning Index (FRI), Working Memory Index (WMI), and Processing Speed Index (PSI). Standardized scores from these domains were used to calculate the Full-Scale Intelligence Quotient (FSIQ). For subsequent analyses, FSIQ was dichotomized, with values <90 defined as below-average cognitive development and values ≥90 defined as normal cognitive level.

The Ages and Stages Questionnaire, Third Edition (ASQ-3), was used to assess neurodevelopmental outcomes in children aged 3 to 6 years. The ASQ-3 is a parent-reported screening tool that evaluates five developmental domains, including communication, gross motor skills, fine motor skills, problem-solving, and personal–social skills. It has been widely applied for the early detection of developmental delays and has demonstrated good structural validity in Chinese pediatric populations [60]. According to the ASQ-3 manual [61], developmental delay in each domain was defined as scores falling below the established cutoff values, corresponding to ≥2 standard deviations below the normative mean.

All assessors received standardized training under the supervision of clinically qualified psychologists. Assessments were conducted in a quiet and private room within the study hospital to minimize environmental disturbances. Data were entered and processed by researchers who were blinded to exposure information, and raw scores were subsequently calculated for each child.

### 2.4. Covariates

Demographic characteristics of pregnant women were collected through face-to-face interviews conducted by trained interviewers using structured questionnaires. The information obtained included maternal age, place of birth, pre-pregnancy body mass index (BMI), occupational status, smoking exposure (including active and passive smoking), alcohol consumption, parity, multiple pregnancy status, and regular physical activity during pregnancy. The pregnant woman self-reported her pre-pregnancy weight and height during her first prenatal appointment after getting pregnant. The pre-pregnancy BMI is calculated by dividing the weight before pregnancy by the square of the height (unit: kilograms per square meter). In addition, information about pregnancy complications, gestational age, infant weight, height and gender was obtained from medical records.

Based on the existing data and literature review, a directed acyclic graph (DAG) (Figure S2) was constructed to represent the assumed causal relationships among variables and to guide the identification of potential confounders for statistical adjustment [62]. Accordingly, the confounding factors adjusted for in all models included prepregnancy BMI, maternal education, high-risk pregnancy, children’s gender, children’s age, gestational age, and delivery way.

### 2.5. Statistical Analysis

Continuous variables with a normal distribution were expressed as the mean (±SD) and compared using the independent sample *t*-test, while continuous variables with a non-normal distribution were expressed as the median (interquartile range, IQR) and compared using the Wilcoxon rank sum test. The categorical variable is expressed as *N* (%) and a chi-square test is used. Given the right-skewed distribution of NEO concentrations in cord plasma, natural logarithm (ln) transformation was applied to achieve approximate normality when NEOs were treated as continuous variables. Spearman correlation coefficients were used to evaluate the correlations among individual NEOs.

Generalized linear models (GLMs) were used to evaluate associations between individual NEOs and neurodevelopmental outcomes. Both unadjusted (crude) and adjusted models were constructed. Unadjusted models were included for exploratory purposes and to facilitate comparison with adjusted estimates, while our primary inferences were based on adjusted models. NEOs concentrations were analyzed as both continuous variables (ln-transformed, per one ln-unit increase) and categorical variables. Based on detection rate, NEOs were categorized using the following strategies. For compounds with detection rates < 50%, concentrations were dichotomized as <LOD and ≥LOD. For detection rates between 50% and 66%, three categories were defined: <LOD, LOD–median, and ≥median. For detection rates >66%, concentrations were categorized into tertiles (T1, T2, T3), representing low, medium, and high exposure group. In all analyses, the lowest exposure category was used as the reference group. Tests for trend were performed by assigning the median concentration of each category and modeling it as a continuous variable. To account for multiple comparisons, false discovery rate (FDR) correction was applied using the Benjamini–Hochberg method.

Simultaneous exposure to multiple pesticides reflects real-life conditions. Therefore, mixture analyses were conducted using Bayesian kernel machine regression (BKMR) and quantile g-computation (qgcomp) to evaluate the joint associations between pesticide mixtures and neurodevelopment. In mixture models, NEOs concentrations were natural log-transformed to reduce skewness and were included as continuous variables without further standardization. Values below LOD were handled as described above. For BKMR, all exposures were included simultaneously using a Gaussian kernel. The model was fitted with 20,000 iterations, which was sufficient to achieve stable estimates based on trace plot inspection. For qgcomp, exposures were modeled as quantiles, and the estimated effect represents the joint change associated with a simultaneous one-quantile increase in all exposures. All analyses were conducted using R software (version 4.2.2), and statistical significance was set at *p* < 0.05.

## 3. Results

### 3.1. Characteristics of the Study Population

This prospective birth cohort study included complete baseline and follow-up data from 114 mother–child pairs. At baseline, 13.16% of mothers were underweight, 71.05% had normal weight, and 15.79% were overweight according to pre-pregnancy BMI classification. Mean maternal age at delivery was 28.73 ± 4.10 years. Among the 114 newborns, 58 (50.88%) were girls and 56 (49.12%) were boys. The mean gestational age was 38.60 ± 1.13 weeks. At follow-up (mean age: 4.61 ± 0.57 years), all children completed WPPSI-IV (CN) and the ASQ. According to the 2005 Chinese National Survey on Physical Growth of Children under Seven Years, 77.20% of the children had a normal BMI, 14.91% were underweight, and 7.19% were classified as overweight or obese (Table 1).

### 3.2. NEOs Concentrations in Cord Plasma

We quantified the concentrations of 10 NEOs in cord plasma. Seven NEOs (ACE, DIN, NACE, CLO, SUL, IMI, and NIT) showed detection frequencies exceeding 50%. DIN had the highest detection rate (96.49%), followed by NACE (85.09%). The highest median concentration was observed for DIN (0.160 ng/mL), followed by NACE (0.073 ng/mL) (Table S1). Pairwise correlations among the ten NEOs were generally weak, with correlation coefficients (r) ranging from −0.13 to 0.31. The strongest positive correlation was observed between CLO and THIA (r = 0.31), followed by ACE and FLU (r = 0.25). Among the negative correlations, the strongest was observed between ACE and TMX (r = −0.13), followed by ACE and NACE (r = −0.11) (Figure S3). Multicollinearity diagnostics using the variance inflation factor (VIF) indicated no significant collinearity among the 10 NEOs (all VIF < 2.0; Table S2).

### 3.3. Assessment Results of Children’s Neurocognitive Function

As shown in Table S3, the mean (±SD) FSIQ score of preschool children (*N* = 114) was 86.08 ± 12.19, ranging from 27 to 118. Among the subscales, the highest mean score was observed in the FRI (99.98 ± 12.30), followed by the PSI (99.18 ± 11.28) and VSI (91.61 ± 11.35). The lowest mean score was observed in the VCI (84.41 ± 13.51). Table S4 summarizes domain-specific scores and the prevalence of subnormal performance. A total of 3.51% of children exhibited communication delay, 7.02% exhibited gross motor delay, 6.14% exhibited fine motor delay, 3.51% exhibited problem-solving delay, and 5.26% exhibited personal–social delay. A statistically significant difference was observed in the communication domain, where the median score was lower in the FSIQ < 90 group (P50 = 50.0) compared with the FSIQ ≥ 90 group (P50 = 52.6). No significant differences were found between the two groups in the other four domains (Table S5).

### 3.4. Associations of Individual NEOs with Neurocognitive Development

In the unadjusted model (Model 1), one-unit increases in ln-transformed concentrations of DIN (β = −3.21, 95% CI: −5.35, −1.08), NACE (β = −2.12, 95% CI: −4.22, −0.02), and CLO (β = −2.92, 95% CI: −5.29, −0.55) were associated with lower FSIQ scores. Children in the high DIN exposure group were associated with lower FSIQ scores compared with those in the low-exposure group (β = −9.47, 95% CI: −15.03, −3.92). After adjustment for covariates (Model 2), inverse associations with FSIQ were observed for ln-transformed DIN (β = −2.04, 95% CI: −4.04, −0.03) and CLO (β = −3.24, 95% CI: −5.37, −1.11). Similarly, higher DIN exposure was associated with lower FSIQ scores compared with the low-exposure group (β = −7.00, 95% CI: −12.77, −1.23). The positive association observed for high ACE exposure in Model 1 was attenuated after adjustment and was no longer statistically significant (Table 2). Consistent patterns were observed for low-average intelligence status. In Model 2, each one-unit increase in ln-transformed DIN concentration was related to higher odds of low-average intelligence (OR = 1.66, 95% CI: 1.05, 2.76). Compared with children in the low-exposure group, those in the high DIN exposure group had higher odds of low-average intelligence (OR = 4.41, 95% CI: 1.38, 15.66), with a significant positive trend across exposure categories (*p*-trend = 0.025) (Table S7).

Associations between NEO exposure and children’s performance on the ASQ were also examined. In the unadjusted models (Model 1), DIN and CLO exposure showed positive associations with fine motor scores (β = 0.03, 95% CI: 0.00, 0.05; β = 0.04, 95% CI: 0.01, 0.07), while FLU was inversely associated (β = −0.04, 95% CI: −0.07, −0.00). After adjustment for covariates (Model 2), these associations were attenuated. However, significant inverse associations emerged for THIA exposure with communication scores (β = −0.07, 95% CI: −0.13, −0.01), IMI exposure with gross motor scores (β = −0.02, 95% CI: −0.04, −0.01), and TMX exposure with both gross motor (β = −0.05, 95% CI: −0.09, −0.01) and fine motor scores (β = −0.06, 95% CI: −0.11, −0.01) (Table 3, Tables S8 and S9).

After correction for multiple comparisons using the FDR method, none of the above associations remained statistically significant.

### 3.5. Combined Effects of NEOs Mixtures

**Figure 1:**
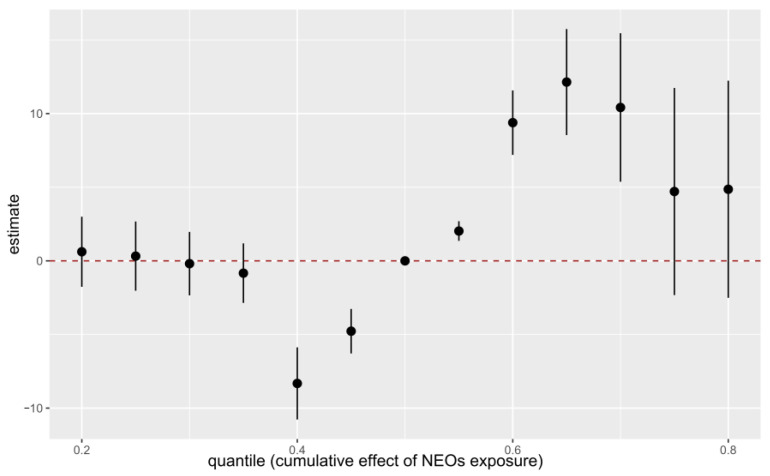
Joint effects of the 10 NEOs on low-average intelligence by the cluster-BKMR model. The dashed horizontal line at y = 0 represents the reference level, indicating no effect. Adjusted for prepregnancy BMI, maternal education, high-risk pregnancy, children’s gender, children’s age, gestational age, delivery way.

The BKMR model (Figure 1) suggested an increasing trend in the posterior risk of low-average intelligence across cumulative NEOs exposure quantiles (0.40 to 0.65), indicating a potential non-linear dose–response relationship. The overall mixture effect appeared to be elevated when all exposures were at or above the 55th percentile compared with their median levels. BKMR analysis examining the association between the NEOs mixture and ASQ scores (Figure S4) provided no clear evidence of a significant overall mixture effect of an overall mixture effect when all exposures were set at selected percentiles compared with their 50th percentile.

Qgcomp analysis for the risk of low-average intelligence (Figure S5) suggested that DIN and SUL had relatively higher positive weight contributions. SUL accounted for approximately 29.20% of the positive weights, while DIN accounted for 50.84%. The overall mixture effect suggested a potential positive association with the risk of low-average intelligence.

For ASQ scores (Figure S6), the contribution patterns varied across domains. IMI appeared to contribute the largest proportion of weights for the communication domain (45.70%). In contrast, TMX was more prominently associated with negative weight contributions in the other domains, including gross motor (60.95%), fine motor (48.46%), problem-solving (54.64%), and personal–social (36.49%).

### 3.6. Stratified Analysis

**Figure 2:**
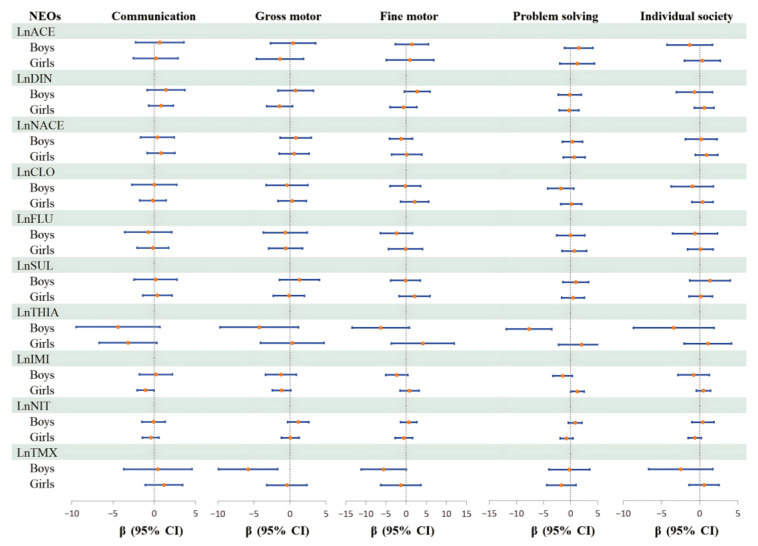
Forest plot of the association between 10 NEOs in cord plasma and ASQ score: stratified by children’s gender. Adjusted for prepregnancy BMI, maternal education, high-risk pregnancy, children’s gender, children’s age, gestational age, delivery way.

Stratified analyses were conducted to examine associations between prenatal NEOs exposure and ASQ scores by gender (Figure 2). Overall, the results suggested potential gender-specific differential associations.

Among girls, IMI exposure was associated with lower communication scores (β = −1.05, 95% CI: −2.09, −0.02). Conversely, IMI exposure was correlated with higher problem-solving scores (β = 1.26, 95% CI: 0.02, 2.50). Among boys, TMX exposure was related to lower gross motor scores (β = −5.79, 95% CI: −9.88, −1.70). Likewise, THIA exposure was associated with lower problem-solving scores (β = −7.66, −11.86, −3.45). These findings may suggest possible differential links between prenatal NEOs exposure and childhood neurodevelopment across genders (Table S10).

## 4. Discussion

To our knowledge, this study comprehensively explored associations between prenatal NEOs exposure measured in umbilical cord plasma and neurocognitive development among preschool children. In single-pollutant models, prenatal DIN and CLO exposure were associated with lower FSIQ scores. THIA showed a suggestive association with reduced communication performance, while IMI was associated with lower gross motor scores. TMX was associated with adverse outcomes in both gross and fine motor domains. Furthermore, NEOs may exert divergent effects on neurodevelopment across gender subgroups. In girls, IMI exposure was associated with lower communication scores. In boys, TMX exposure was associated with lower gross motor performance, and THIA was associated with lower problem-solving scores.

To date, very few studies have reported NEO concentrations in fetal umbilical cord plasma. A study from Jiangsu Province, China [63], reported NEOs exposure levels in maternal serum during early pregnancy. The detection rates of IMI, TMH, and CLO (53.5–78%) in that study were comparable to those found in our study, but the exposure levels were lower with all median concentrations being 0.01 ng/mL, and the detection rate of ACE was less than 5%, with median values below the method detection limit. In contrast, our study found that the median concentrations of IMI, ACE, and CLO were higher (0.038 ng/mL, 0.027 ng/mL, 0.015 ng/mL). This difference may be attributed to greater NEOs accumulation in pregnant women as pregnancy progresses from early to late gestation. A study conducted in Wuxi, China [64], reported that DIN was not detected in any serum samples, while detection rates of the other 6 NEOs (ACE, CLO, THIA, IMI, TMX, and NIT) were substantially lower than those observed in our study (4.2–28.3%). These differences may be related to regional variations. Previous studies have indicated that NEOs contamination is more severe in the Yangtze River and Pearl River basins in China, with particularly high levels reported in drinking water in provincial capitals of Guangdong and Guangxi [65,66]. Given that humans can be exposed to NEOs through multiple pathways, including food, drinking water, and air, differences in lifestyle and living environments may also contribute to variations in exposure levels across populations [67]. In addition, differences in sample collection, storage, and analytical methods may also partly explain these discrepancies [68].

Numerous animal experiments have demonstrated that NEOs have neurotoxic effects. During the critical windows of neural development, even brief or very low levels of NEOs exposure may lead to neurobehavioral and neurotoxic outcomes, including persistent behavioral alterations [60] and impaired brain function [69,70]. However, to our knowledge, there is a lack of direct evidence showing that NEOs affect children’s neurocognitive development at the mechanistic level. A possible explanation is that intrauterine exposure to NEOs may interfere with fetal neurocognitive development by inducing oxidative stress and immune and inflammatory responses. NEOs can cross the placenta and activate nAChRs [71], thereby inducing oxidative stress and cell death, which may impair nutrient and oxygen transport to the fetus [64,72,73]. They also disrupt immune homeostasis at the maternal-fetal interface by modulating inflammatory mediators and immune cell function [74,75].

This study suggested potential negative associations between 5 NEOs (DIN, CLO, THIA, IMI, and TMX) and children’s neurocognitive development. However, none of these associations remained statistically significant after false discovery rate (FDR) correction. Several associations identified in single-exposure generalized linear models were substantially attenuated or became non-significant in the mixture models. A similar pattern has been reported in our previous study on prenatal neonicotinoid exposure and child health outcomes [76]. Several factors may explain these discrepancies. First, this study involved a large number of statistical comparisons, encompassing multiple exposure metrics, neurodevelopmental outcomes, and modeling approaches (e.g., single-pollutant, multi-pollutant, mixture, and sex-stratified analyses). Such extensive testing increases the likelihood of chance findings. Second, mixture models simultaneously incorporate multiple exposures, thereby better accounting for mutual confounding and generally yielding more conservative effect estimates. Finally, the relatively small sample size (*N* = 114), particularly in stratified and mixture analyses, limits statistical power, which may result in unstable estimates and reduced ability to detect true associations. Taken together, these factors indicate that the observed associations should be interpreted with caution, and that disproportionate emphasis on isolated statistically significant findings should be avoided. Previous epidemiological studies have reported that prenatal exposure to neonicotinoids is associated with a range of adverse neurodevelopmental outcomes, including anencephaly [49], neural tube defects [48], memory impairment [47], and autism spectrum disorder [77]. Our findings are generally consistent with a population-based study reporting a negative association between prenatal neonicotinoid exposure and full-scale IQ in 7-year-old children [48]. Furthermore, experimental studies have suggested that the toxicity of neonicotinoid mixtures may not exceed that of individual compounds [78]. Given the limited evidence regarding the combined effects of neonicotinoid exposure, further well-designed prospective cohort studies and mechanistic investigations are warranted to better elucidate these relationships.

This study has several strengths. First, as a prospective cohort study, it provides a stronger basis for causal inference when assessing the association between NEO exposure and neurocognitive development. Second, most previous studies have relied on urine samples to assess NEOs exposure, while the use of fetal umbilical cord plasma samples in this study better reflects internal exposure levels in the fetus. Finally, compared with other studies that use a single scale, two scales were used to evaluate the neurocognitive development outcomes of children, allowing for a more comprehensive characterization of neurodevelopment.

However, several limitations should also be acknowledged. First, although multiple confounders were adjusted for, residual confounding may still exist. In particular, parental IQ, an important determinant, was not directly measured and was instead approximated using maternal education level, which may not fully capture its effect. Second, the relatively small sample size may result in limited statistical power and increase the risk of model instability and overfitting. Finally, the limit of quantification (LOQ) was not calculated, which may affect the accurate characterization of low-level exposure to NEOs.

In conclusion, this study suggests a potential association between prenatal exposure to neonicotinoid pesticides and children’s neurocognitive development. However, due to limitations such as multiple comparisons, small sample size, and inconsistencies across models, these findings should be regarded as exploratory. Future studies with larger sample sizes, more refined exposure assessment, and improved mixture modeling approaches are warranted to validate these findings.

## 5. Conclusions

Prenatal exposure to NEOs (DIN, CLO, THIA, IMI, TMX) may be negatively associated with neurocognitive development in preschool children. This study provides new epidemiological evidence linking neonicotinoid exposure to children’s neurodevelopment, supporting targeted preventive strategies for reducing neurodevelopmental disorder risks. Future research should focus on combined exposure effects of neonicotinoids and expand the study population to verify these findings.

## Figures and Tables

**Table 1:** The demographic characteristics of the mother–preschool children pairs (*N* = 114).

Variables	Mean ± SD or *N* (%) *
Household registration	
Rural	95 (83.33)
Urban	19 (16.67)
Prepregnancy BMI (kg/m^2^)	
<18.5	15 (13.16)
18.5–23.9	81 (71.05)
≥24	18 (15.79)
Maternal age (y)	28.73 ± 4.10
≤24	22 (19.30)
25–29	46 (40.35)
30–34	31 (27.19)
≥35	15 (13.16)
Smoking and passive smoking	
No	57 (50.00)
Yes	57 (50.00)
Maternal education	
No tertiary education	66 (57.89)
Tertiary education	48 (42.11)
Annual household income	
<6000	44 (38.60)
6000–15,000	47 (41.23)
>15,000	23 (20.18)
High-risk pregnancy	
No	64 (56.14)
Yes	50 (43.86)
Delivery way	
Spontaneous labor	83 (72.81)
Cesarean section	31 (27.19)
Gestational age (weeks)	38.60 ± 1.13
Birth Weight (g)	3111.93 ± 440.50
Birth Height (cm)	49.62 ± 1.98
Gender of children	
Boys	56 (49.12)
Girls	58 (50.88)
Children Age (y)	4.61 ± 0.57
<4	23 (20.18)
4–5	61 (53.51)
>5	30 (26.31)
Weight (kg)	15.92 ± 2.51
Height (cm)	103.64 ± 6.77
BMI of children (kg/m^2^)	
Underweight	17 (14.91)
Normal weight	88 (77.20)
Overweight/Obesity	9 (7.19)

Abbreviations: * shows the mean ± standard deviation; *N* (%), number (percent).

**Table 2:** The Generalized Linear Model (GLM) results of 10 cord plasma NEOs and FSIQ scores in preschool children (*N* = 114).

NEOs (ng/mL)	*N* (%)	Model 1 ^a^		Model 2 ^b^		
		β (95% CI)	*p* Value	β (95% CI)	*p* Value	FDR
LnACE		1.69 (−1.63, 5.00)	0.315	2.74 (−0.47, 5.95)	0.094	0.763
Low-exposure group	38 (33.33)	Ref		Ref		
Medium-exposure group	38 (33.33)	1.55 (−4.16, 7.27)	0.590	0.23 (−5.54, 6.00)	0.937	
High-exposure group	38 (33.34)	5.24 (0.83, 9.65)	0.021	−1.61 (−7.45, 4.23)	0.583	
*p*-trend			0.061		0.540	
LnDIN		−3.21 (−5.35, −1.08)	0.004	−2.04 (−4.04, −0.03)	0.046	0.140
Low-exposure group	38 (33.33)	Ref		Ref		
Medium-exposure group	38 (33.33)	−4.74 (−9.64, 0.16)	0.058	−2.38 (−7.37, 2.62)	0.345	
High-exposure group	38 (33.34)	−9.47 (−15.03, −3.92)	0.001	−7.00 (−12.77, −1.23)	0.018	
*p*-trend			<0.001		0.011	
LnNACE		−2.12 (−4.22, −0.02)	0.048	0.15 (−2.00, 2.29)	0.893	0.763
Low-exposure group	38 (33.33)	Ref		Ref		
Medium-exposure group	38 (33.33)	0.89 (−4.58, 6.37)	0.746	2.14 (−3.01, 7.30)	0.409	
High-exposure group	38 (33.34)	−3.34 (−9.03, 2.35)	0.245	2.46 (−3.58, 8.50)	0.419	
*p*-trend			0.234		0.458	
LnCLO		−2.92 (−5.29, −0.55)	0.016	−3.24 (−5.37, −1.11)	0.003	0.060
Low-exposure group	38 (33.33)	Ref		Ref		
Medium-exposure group	38 (33.33)	0.08 (−4.68, 4.84)	0.974	0.64 (−3.72, 5.00)	0.771	
High-exposure group	38 (33.34)	−2.05 (−7.78, 3.67)	0.477	−1.38 (−6.95, 4.19)	0.622	
*p*-trend			0.465		0.345	

Abbreviations: β, coefficient of regression; CI, Confidence Interval; FDR, false discovery rate. ^a^ unadjusted. ^b^ adjusted for prepregnancy BMI, maternal education, high-risk pregnancy, children’s gender, children’s age, gestational age, delivery way.

**Table 3:** The GLM results of 10 NEOs and ASQ scores (continuous variable) in preschool children (*N* = 114).

NEOs (ng/mL)	Gross Motor				Fine Motor			
	Model 1 ^a^		Model 2 ^b^		Model 1 ^a^		Model 2 ^b^	
	β (95% CI)	*p* Value	β (95% CI)	*p* Value	β (95% CI)	*p* Value	β (95% CI)	*p* Value
LnACE	−0.00 (−0.04, 0.04)	0.987	0.01 (−0.03, 0.05)	0.658	0.00 (−0.04, 0.04)	0.836	0.03 (−0.01, 0.07)	0.184
LnDIN	0.00 (−0.02, 0.03)	0.876	−0.01 (−0.03, 0.02)	0.492	0.03 (0.00, 0.05)	0.042	0.02 (−0.01, 0.05)	0.144
LnNACE	0.01 (−0.01, 0.04)	0.288	0.01 (−0.02, 0.03)	0.571	0.00 (−0.02, 0.03)	0.892	−0.01 (−0.04, 0.02)	0.589
LnCLO	−0.01 (−0.03, 0.02)	0.687	−0.01 (−0.04, 0.02)	0.450	0.04 (0.01, 0.07)	0.004	0.03 (−0.00, 0.06)	0.061
LnFLU	−0.02 (−0.05, 0.02)	0.317	−0.01 (−0.05, 0.02)	0.407	−0.04 (−0.07, −0.00)	0.035	−0.03 (−0.07, 0.01)	0.118
LnSUL	0.00 (−0.03, 0.03)	0.857	0.01 (−0.02, 0.04)	0.635	0.02 (−0.01, 0.06)	0.127	0.03 (−0.00, 0.06)	0.057
LnTHIA	−0.03 (−0.09, 0.03)	0.316	−0.04 (−0.11, 0.02)	0.164	0.00 (−0.06, 0.06)	0.946	−0.02 (−0.09, 0.05)	0.589
LnIMI	−0.02 (−0.03, 0.00)	0.112	−0.02 (−0.04, −0.01)	0.038	−0.00 (−0.02, 0.02)	0.701	−0.01 (−0.03, 0.01)	0.512
LnNIT	0.01 (−0.01, 0.03)	0.237	0.01 (−0.00, 0.03)	0.095	0.01 (−0.01, 0.02)	0.503	0.00 (−0.01, 0.02)	0.616
LnTMX	−0.04 (−0.08, 0.01)	0.100	−0.05 (−0.09, −0.01)	0.024	−0.04 (−0.09, 0.00)	0.070	−0.06 (−0.11, −0.01)	0.011

Abbreviations: CI, Confidence Interval. ^a^ unadjusted. ^b^ adjusted for prepregnancy BMI, maternal education, high-risk pregnancy, children’s gender, children’s age, gestational age, delivery way.

## Data Availability

The raw data supporting the conclusions of this article will be made available by the authors on request.

## Supplementary Materials

The following supporting information can be downloaded at: https://www.mdpi.com/article/10.3390/toxics14050445/s1, Figure S1: Representative chromatograms of NEOs standards. (A) Dinotefuran (DIN) standard; (B) Acetamiprid (ACE) standard. Peaks were acquired under UPLC-MS/MS multiple reaction monitoring (MRM) mode. (C) N-desmethyl-acetamiprid (NACE) standard; (D) Clothianidin (CLO) standard. (E) Flupyradifurone (FLU), Sulfoxaflor (SUL) and Thiacloprid (THIA) standards; (F) Imidacloprid (IMI) standard. (G) Nitenpyram (NIT) standard; (H) Thiamethoxam (TMX) standard; Figure S2: Directed acyclic graph (DAG) of NEOs exposure and child neurocognitive development. Nodes represent exposure, outcome, and covariates (confounders). Yellow circle with a right-pointing triangle: Primary exposure variable (NEOs). Blue circle with a vertical line: Outcome variable (children’s neurological and cognitive development). Red/pink nodes: Ancestors of both exposure and outcome. Yellow nodes: Ancestors of the exposure. Blue nodes: Ancestors of the outcome. Directed edges represent hypothesized causal relationships based on published literature and expert knowledge, not positive/negative statistical associations. Edge colors: Green edges indicate causal paths; magenta/pink edges indicate biasing paths; Figure S3: Correlations among the 10 NEOs measured in cord plasma. Significance levels: * *p* < 0.05, ** *p* < 0.01, *** *p* < 0.001; Figure S4: Joint effects of the 10 NEOs on the preschool education and ASQ score by BKMR model. (A) The BKMR result of communication score; (B) The BKMR result of gross motor score; (C) The BKMR result of fine motor scoring; (D) The BKMR result of problem solving score; (E) The BKMR result of individual-society scoring; Figure S5: The Qgcomp effect map of 10 NEOs combined with low-average intelligence. Adjusted for prepregnancy BMI, maternal education, high-risk pregnancy, children’s gender, children’s age, gestational age, delivery way. The area corresponding to each substance represents its weight contribution to the overall mixture effect in the positive or negative direction; Figure S6: The Qgcomp effect map of 10 NEOs combined with ASQ score. (A) The Qgcomp result of communication score; (B) The Qgcomp result of gross motor score; (C) The Qgcomp result of fine motor scoring; (D) The Qgcomp result of problem solving score; (E) The Qgcomp result of individual-society scoring; Table S1: Detection frequency and concentration distribution of NEOs in cord plasma; Table S2: Diagnosis of multicollinearity among the 10 NEOs in cord plasma; Table S3: The WPPSI-IV (CN) result for preschool children (*N* = 114); Table S4: The ASQ result for preschool children (*N* = 114); Table S5: The analysis of the five ASQ domain scores was stratified by FSIQ (*N* = 114); Table S6: The Generalized Linear Model (GLM) results of cord plasma NEOs and FSIQ score in preschool children (*N* = 114); Table S7: Association between cord plasma NEOs levels and risk of low-average intelligence (*N* = 114); Table S8: Correlation between cord plasma NEOs concentration (ng/mL) and ASQ score (*N* = 114) (unadjusted); Table S9: Correlation between cord plasma NEOs concentration (ng/mL) and ASQ score (*N* = 114) (adjusted); Table S10: Stratified analysis of 10 cord plasma NEOs and ASQ scores; Table S11: Interaction analysis of 10 cord plasma NEOs and ASQ scores.
