# Supplementary material for: Prenatal Exposure to Neonicotinoid Insecticides and Neurological and Cognitive Development in Preschool Children: Evidence from a Birth Cohort in Guangxi, China
Source: Toxics. 2026 May 20;14(5):445. doi: 10.3390/toxics14050445 (PMC13211703; doi:10.3390/toxics14050445)
Supplement: Supplementary file 1 [file toxics-14-00445-s001.zip › toxics-4254643-supplementary.pdf]

Supplementary Material for

Prenatal Exposure to Neonicotinoid Insecticides and Neurological and Cognitive Development in Preschool Children: Evidence from a Birth Cohort in Guangxi, China

Submitted by

Qingqing Liang [1,†], Haiyan Li [1,†], Lihong Zhou [1], Changhui Mu [2], Mengrui Lin [2], Qian Liao [1], Shun Liu [3], Xiaoqiang Qiu [1], Dongping Huang [2], Dongxiang Pan [1,4,*] and Xiaoyun Zeng [1,5,*]

[1] Department of Epidemiology and Health Statistics, School of Public Health, Guangxi Medical University, Nanning 530021, China; liangqingqing@sr.gxmu.edu.cn (Q.L.); lhy@sr.gxmu.edu.cn (H.L.); 18277103236@163.com (L.Z.); maaiya@163.com (Q.L.); xqqiu9999@163.com (X.Q.)
[2] Department of Sanitary Inspection, School of Public Health, Guangxi Medical University, Nanning 530021, China; everyday202605@163.com (C.M.); linmengrui1996@163.com (M.L.); dongpinghuang@gxmu.edu.cn (D.H.)
[3] Department of Maternal, Child and Adolescent Health, School of Public Health, Guangxi Medical University, Nanning 530021, China; liushun@gxmu.edu.cn
[4] China (Guangxi)-ASEAN Engineering Research Center of Big Data for Public Health, Guangxi Medical University, Nanning 530021, China
[5] Department of Epidemiology and Health Statistics, School of Public Health, Guilin Medical University, No. 1 Zhiyuan Road, Lingui District, Guilin 541199, China
* Correspondence: gxpandongxiang@163.com (D.P.); zengxiaoyun@gxmu.edu.cn (X.Z.)

Texts: 0.
Figures: 6.
Tables: 11.

Contents:

Figure S1. Representative chromatograms of NEOs standards.

Figure S2. Directed acyclic graph (DAG) of NEOs exposure and child neurocognitive development.

Figure S3. Correlations among the 10 NEOs measured in cord plasma.

Figure S4. Joint effects of the 10 NEOs on the preschool education and ASQ score by BKMR model.

Figure S5. The Qgcomp effect map of 10 NEOs combined with low-average intelligence.

Figure S6. The Qgcomp effect map of 10 NEOs combined with ASQ score.

Table S1. Detection frequency and concentration distribution of NEOs in cord plasma.

Table S2. Diagnosis of multicollinearity among the 10 NEOs in cord plasma.

Table S3. The WPPSI-IV (CN) result for preschool children (*N* = 114).

Table S4. The ASQ result for preschool children (*N* = 114).

Table S5. The analysis of the five ASQ domain scores was stratified by FSIQ (*N* = 114).

Table S6. The Generalized Linear Model (GLM) results of cord plasma NEOs and FSIQ score in preschool children (N = 114).

Table S7. Association between cord plasma NEOs levels and risk of low-average intelligence (*N* = 114).

Table S8. Correlation between cord plasma NEOs concentration (ng/mL) and ASQ score (*N* = 114) (unadjusted).

Table S9. Correlation between cord plasma NEOs concentration (ng/mL) and ASQ score (*N* = 114) (adjusted).

Table S10. Stratified analysis of 10 cord plasma NEOs and ASQ scores.

Table S11. Interaction analysis of 10 cord plasma NEOs and ASQ scores.

(A)

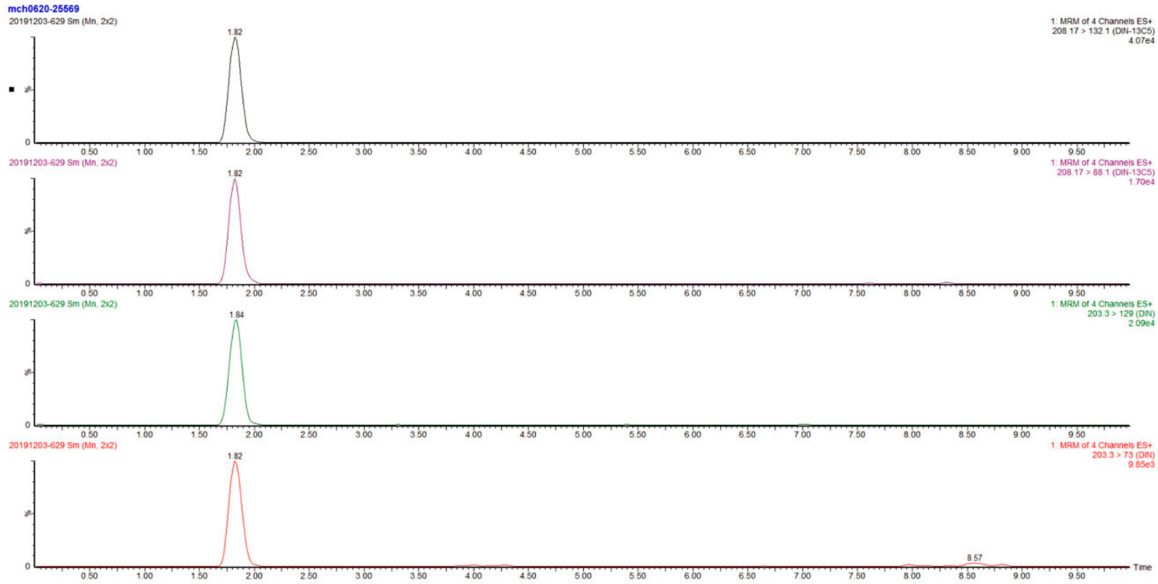
mch0620-25569
20191203-629 Sm (Mn, 2x2)
1: MRM of 4 Channels ES+
208.17 > 132.1 (DIN-13C5)
4.07e4
1.82
208.17 > 88.1 (DIN-13C5)
1.70e4
203.3 > 129 (DIN)
2.09e4
1.84
203.3 > 73 (DIN)
9.85e3
8.57
Time

(B)

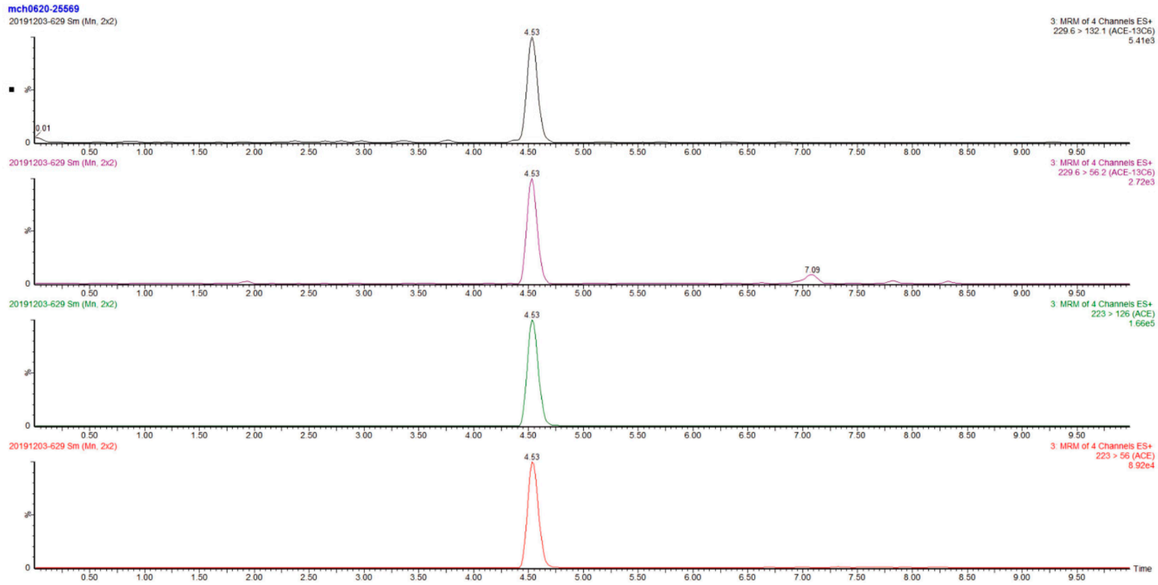
mch0620-25569
20191203-629 Sm (Mn, 2x2)
3: MRM of 4 Channels ES+
229.6 > 132.1 (ACE-13C6)
5.41e3
4.53
0.01
229.6 > 56.2 (ACE-13C6)
2.72e3
7.09
223 > 126 (ACE)
1.66e5
223 > 56 (ACE)
8.92e4
Time

(C)

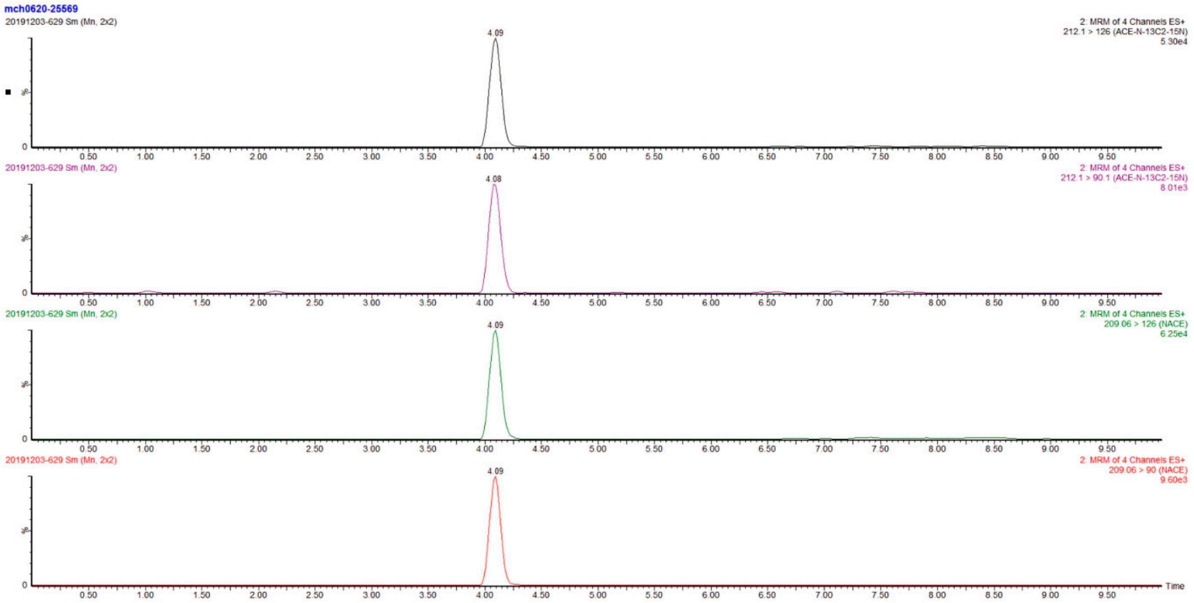
mch0620-25569
20191203-629 Sm (Mn, 2x2)
2: MRM of 4 Channels ES+
212.1 > 126 (ACE-N-13C2-15N)
5.30e4
2: MRM of 4 Channels ES+
212.1 > 90.1 (ACE-N-13C2-15N)
8.01e3
2: MRM of 4 Channels ES+
209.06 > 126 (NACE)
6.25e4
2: MRM of 4 Channels ES+
209.06 > 90 (NACE)
9.60e3
Time

(D)

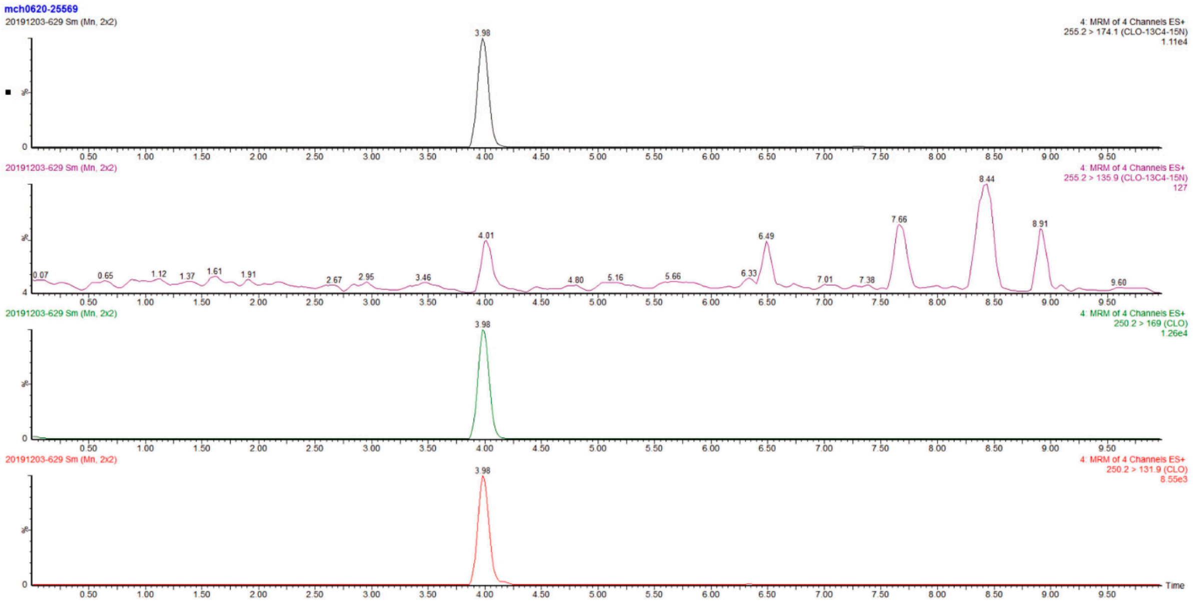
mch0620-25569
20191203-629 Sm (Mn, 2x2)
4: MRM of 4 Channels ES+
255.2 > 174.1 (CLO-13C4-15N)
1.11e4
4: MRM of 4 Channels ES+
255.2 > 135.9 (CLO-13C4-15N)
127
4: MRM of 4 Channels ES+
250.2 > 169 (CLO)
1.26e4
4: MRM of 4 Channels ES+
250.2 > 131.9 (CLO)
8.55e3
Time

(E)

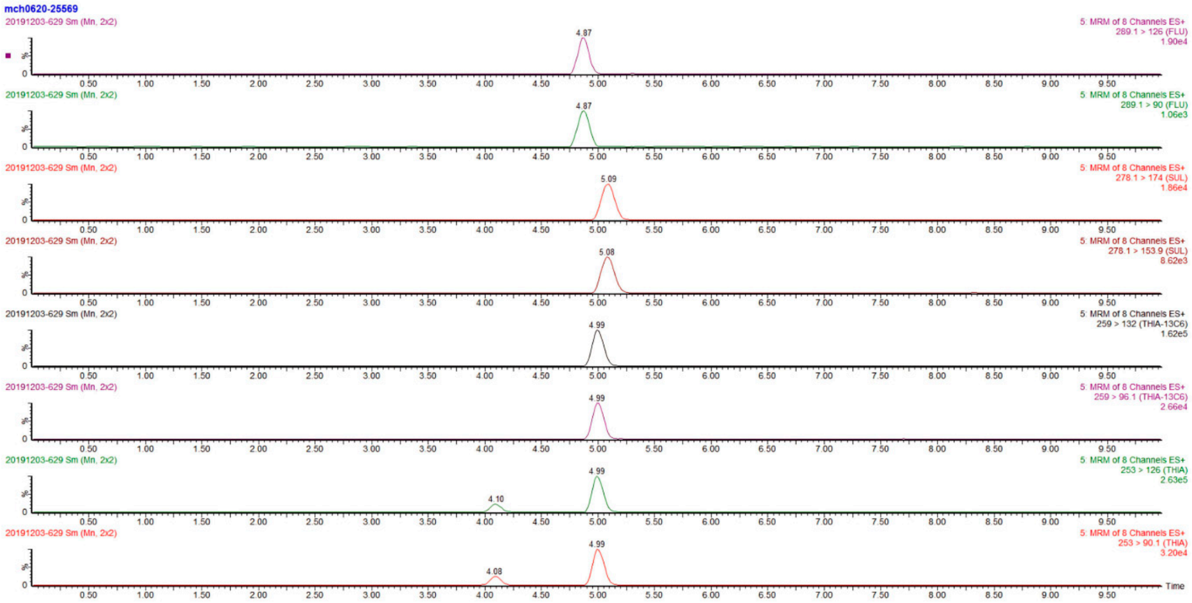

(F)

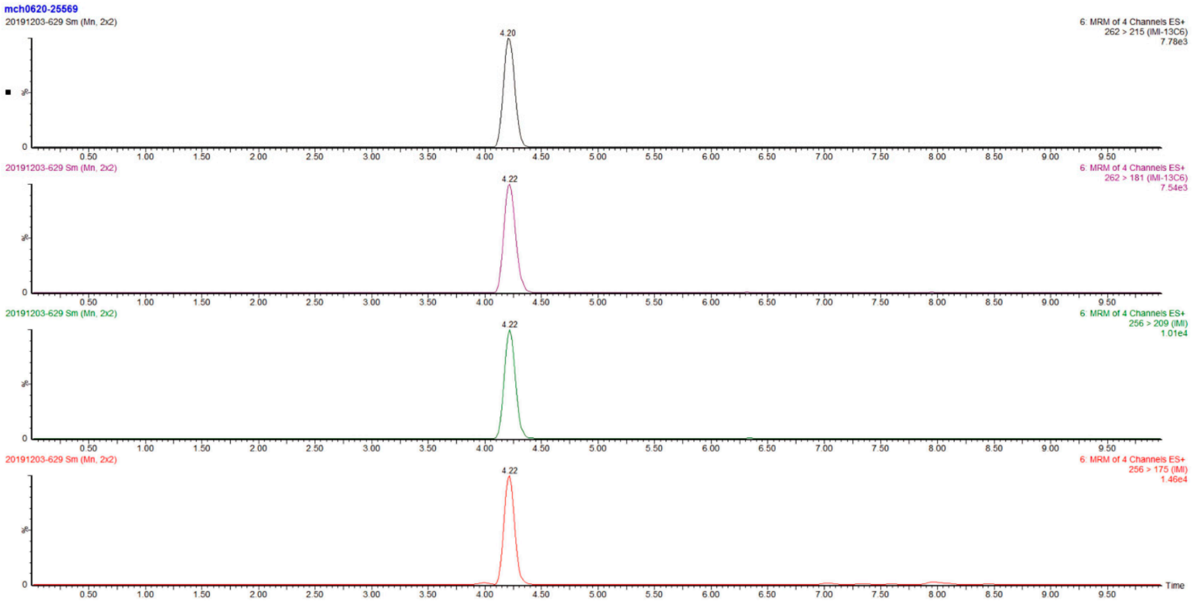

(G)

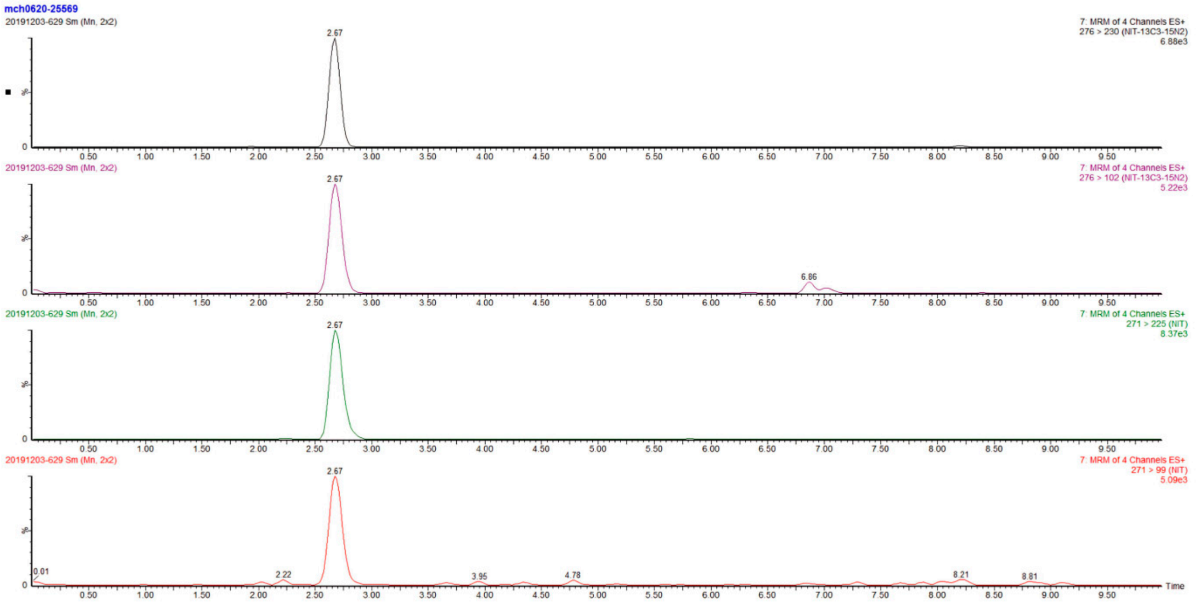

(H)

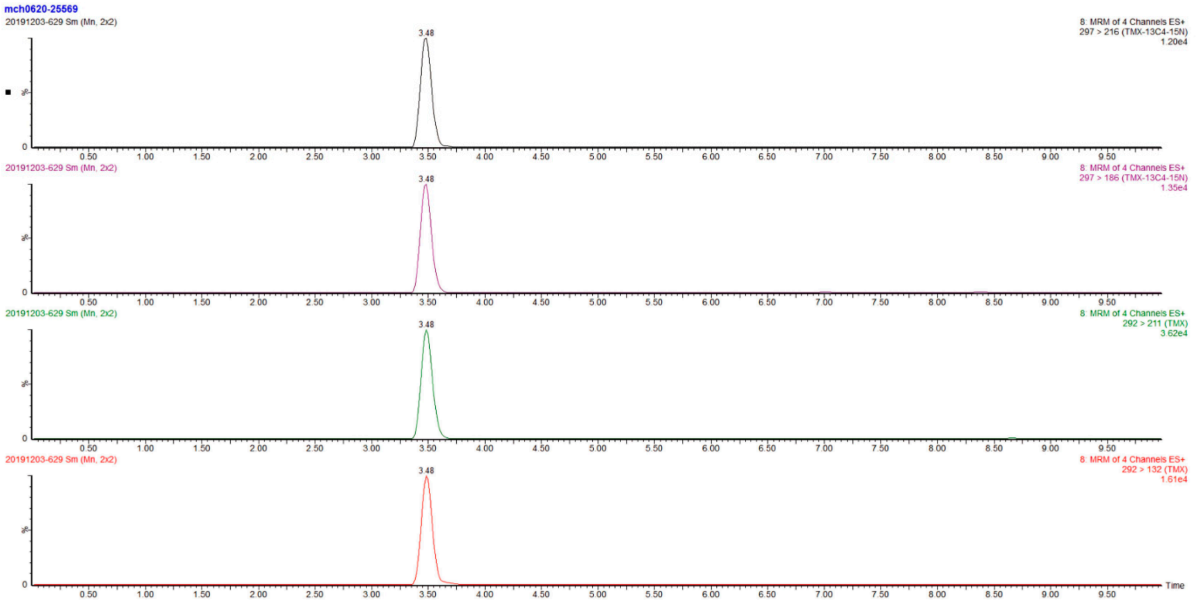

Figure S1. Representative chromatograms of NEOs standards. (A) Dinotefuran (DIN) standard; (B) Acetamiprid (ACE) standard. Peaks were acquired under UPLC-MS/MS multiple reaction monitoring (MRM) mode. (C) N-desmethyl-acetamiprid (NACE) standard; (D) Clothianidin (CLO) standard. (E) Flupyradifurone (FLU), Sulfoxaflor (SUL) and Thiacloprid (THIA) standards; (F) Imidacloprid (IMI) standard. (G) Nitenpyram (NIT) standard; (H) Thiamethoxam (TMX) standard.

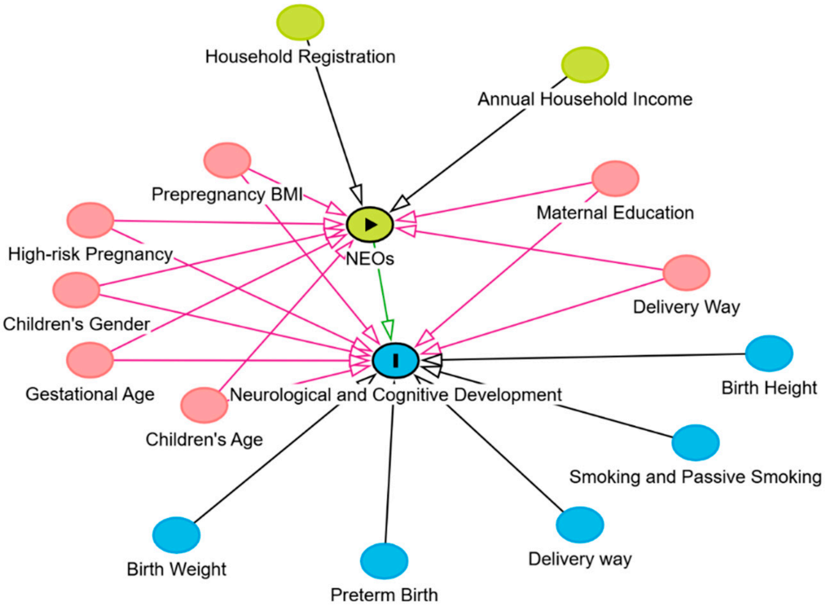

Figure S2. Directed acyclic graph (DAG) of NEOs exposure and child neurocognitive development. Nodes represent exposure, outcome, and covariates (confounders). Yellow circle with a right-pointing triangle: Primary exposure variable (NEOs). Blue circle with a vertical line: Outcome variable (children's neurological and cognitive development). Red/pink nodes: Ancestors of both exposure and outcome. Yellow nodes: Ancestors of the exposure. Blue nodes: Ancestors of the outcome. Directed edges represent hypothesized causal relationships based on published literature and expert knowledge, not positive/negative statistical associations. Edge colors: Green edges indicate causal paths; magenta/pink edges indicate biasing paths.

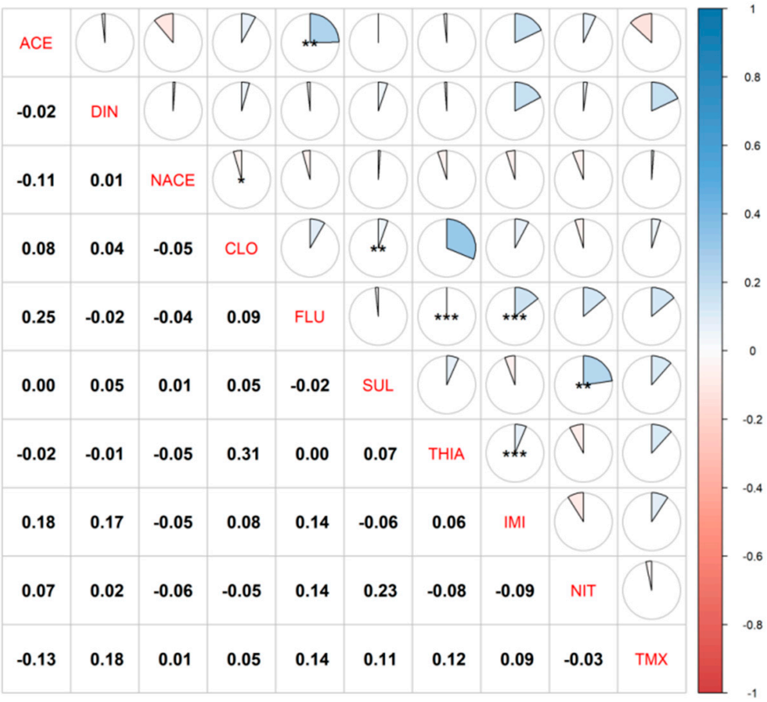

Figure S3. Correlations among the 10 NEOs measured in cord plasma. Significance levels: * *p* <0.05, ** *p* <0.01, *** *p* <0.001.

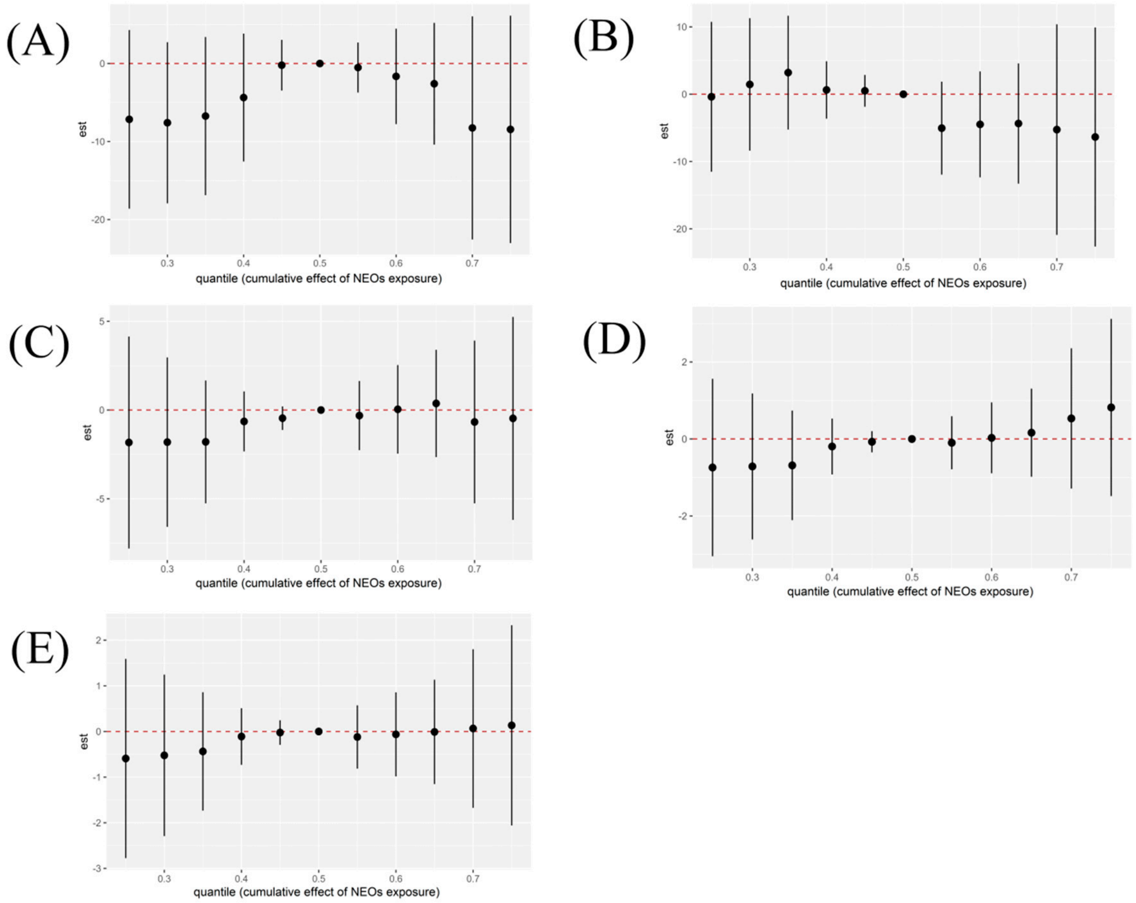

Figure S4. Joint effects of the 10 NEOs on the preschool education and ASQ score by BKMR model. (A)The BKMR result of communication score; (B)The BKMR result of gross motor score; (C)The BKMR result of fine motor scoring; (D)The BKMR result of problem solving score; (E)The BKMR result of individual-society scoring.

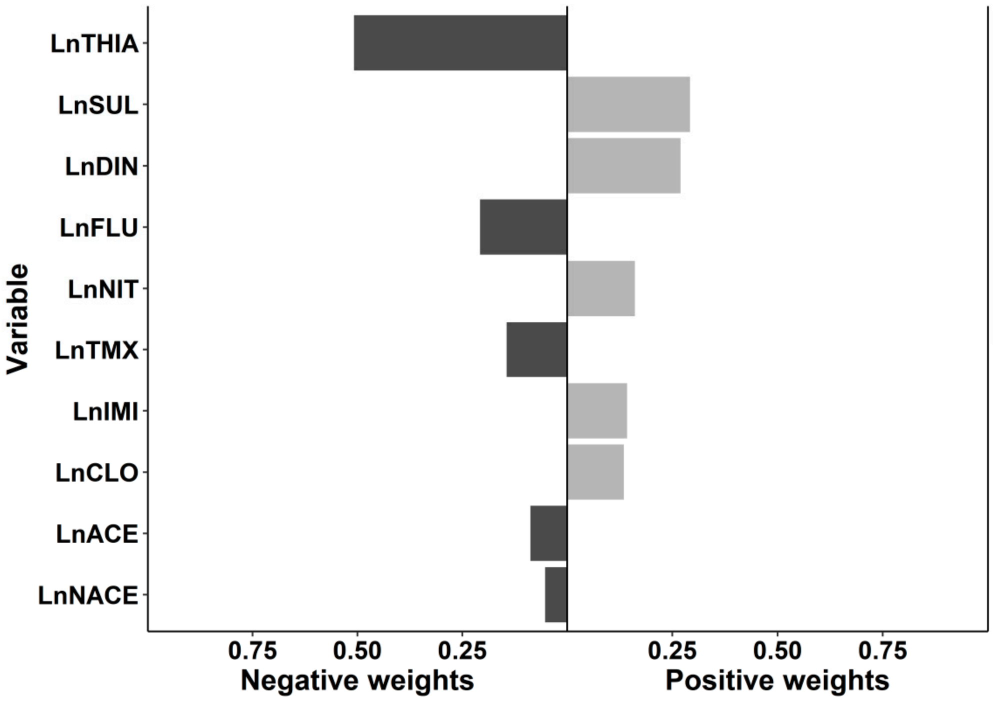

Figure S5. The Qgcomp effect map of 10 NEOs combined with low-average intelligence. Adjusted for prepregnancy BMI, maternal education, high-risk pregnancy, children's gender, children's age, gestational age, delivery way. The area corresponding to each substance represents its weight contribution to the overall mixture effect in the positive or negative direction.

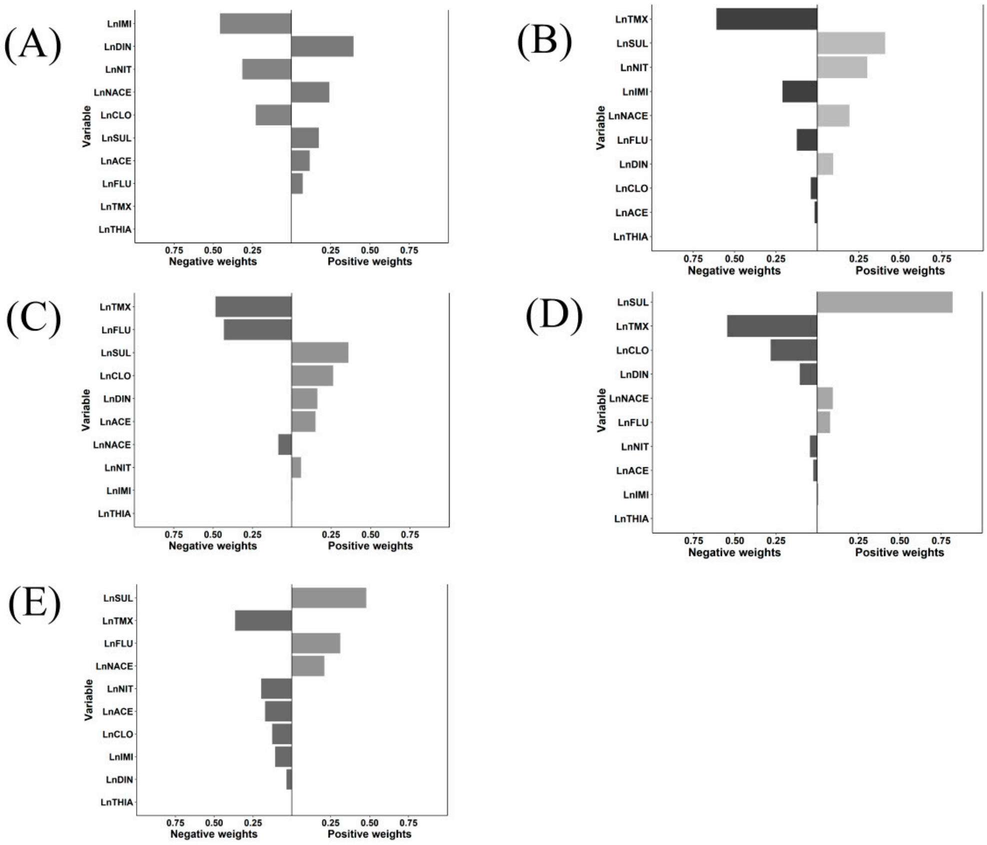

Figure S6 The Qgcomp effect map of 10 NEOs combined with ASQ score. (A)The Qgcomp result of communication score; (B)The Qgcomp result of gross motor score; (C)The Qgcomp result of fine motor scoring; (D)The Qgcomp result of problem solving score; (E)The Qgcomp result of individual-society scoring.

Table S1. Detection frequency and concentration distribution of NEOs in cord plasma.

NEOs	P25 (ng/mL)	P50 (ng/mL)	P75 (ng/mL)	P95 (ng/mL)	IQR (ng/mL)	Detection rate (%)
ACE	0.014	0.015	0.019	0.035	0.005	84.21
DIN	0.075	0.160	0.305	0.948	0.228	96.49
NACE	0.034	0.073	0.157	0.552	0.123	85.09
CLO	0.015	0.027	0.043	0.112	0.028	75.44
FLU	＜LOD	＜LOD	0.056	0.112	0.038	30.70
SUL	＜LOD	0.036	0.077	0.192	0.060	62.28
THIA	＜LOD	＜LOD	＜LOD	0.025	0.010	15.79
IMI	＜LOD	0.038	0.096	0.483	0.087	71.93
NIT	＜LOD	0.067	0.156	0.783	0.147	70.18
TMX	＜LOD	＜LOD	0.033	0.072	0.015	49.12

Abbreviations: P25, 25th percentile; P50, median (50th percentile); P75, 75th percentile; P95, 95th percentile; IQR: interquartile range.

Table S2. Diagnosis of multicollinearity among the 10 NEOs in cord plasma.

NEOs	VIF	Tolerance
ACE	1.39	0.720
DIN	1.10	0.912
NACE	1.19	0.839
CLO	1.24	0.808
FLU	3.17	0.316
SUL	1.24	0.806
THIA	2.57	0.389
IMI	2.17	0.461
NIT	1.13	0.888
TMX	1.07	0.936

Abbreviations: VIF, variance inflation factor; VIF <5 and tolerance >0.2 suggest no severe multicollinearity.

Table S3. The WPPSI-IV (CN) result for preschool children (N = 114).

Variables	Mean ± SD	Min	P25	P50	P75	Max
FSIQ	86.08 ± 12.19	27	79.00	85.00	92.50	118.00
VCI	84.41 ± 13.51	36	77.00	83.00	91.50	117.00
VSI	91.61 ± 11.35	50	83.75	91.00	97.00	127.00
FRI	99.98 ± 12.30	50	92.00	102.00	109.75	123.00
WMI	90.37 ± 13.08	42	82.00	90.00	100.00	121.00
PSI	99.18 ± 11.28	55	94.25	100.00	105.00	133.00

Abbreviations: FSIQ, Full-Scale Intelligence Quotient; VCI, Verbal Comprehension Index; VSI, Visual Spatial Index; FRI, Fluid Reasoning Index; WMI, Working Memory Index; PSI, Processing Speed Index.

Table S4. The ASQ result for preschool children (N = 114).

ASQ	Min	P25	P50	P75	Max	Mean	Subnormal *N* (%)
Communication	25	50	53	60	60	52.83	4 (3.51%)
Gross movement	25	54	55	60	60	54.25	8 (7.02%)
Fine movement	10	40	44	50	60	44.38	7 (6.14%)
Problem solving	25	50	52	60	60	52.21	4 (3.51%)
Individual-society	25	55	55	60	60	54.91	6 (5.26%)

Abbreviations: ASQ, Age and Development Questionnaire; Min, minimum; P25, 25th percentile; P50, median (50th percentile); P75, 75th percentile; Max, maximum; *N*, number; Subnormal, If the score of a subfield is below the critical value and is 2 standard deviations (SD) lower than the mean, it is determined to be abnormal development.

Table S5. The analysis of the five ASQ domain scores was stratified by FSIQ (N = 114).

Variable	Group	*N*	Min	P25	P50	P75	Max	IQR	*p* value*
Communication	FSIQ <90	76	76	25	50.0	52.6	55.0	5.0	<0.001
	FSIQ ≥90	38	38	40	52.6	55.0	60.0	7.4	
Gross Movement	FSIQ <90	76	76	25	54.0	57.5	60.0	6.0	0.351
	FSIQ ≥90	38	38	30	54.0	55.0	60.0	6.0	
Fine Movement	FSIQ <90	76	76	10	40.0	44.1	51.2	11.2	0.212
	FSIQ ≥90	38	38	10	44.1	45.0	50.0	5.9	
Problem Solving	FSIQ <90	76	76	25	50.0	52.2	60.0	10.0	0.278
	FSIQ ≥90	38	38	35	50.0	52.2	60.0	10.0	
Individual Society	FSIQ <90	76	76	25	54.7	55.0	60.0	5.3	0.850
	FSIQ ≥90	38	38	30	54.7	55.0	60.0	5.3	

Abbreviations: * Between-group statistical significance was assessed using the Wilcoxon signed-rank test. ASQ, Age and Development Questionnaire; FSIQ, Full-Scale Intelligence Quotient; Min, minimum; P25, 25th percentile; P50, median (50th percentile); P75, 75th percentile; Max, maximum; IQR: interquartile range; *N*, number.

Table S6. The Generalized Linear Model (GLM) results of cord plasma NEOs and FSIQ score in preschool children (N = 114).

NEOs (ng/mL)	*N* (%)	Model 1[a]		Model 2[b]		
		**β (95% CI)**	***p* value**	**β (95% CI)**	***p* value**	***FDR***
LnACE		1.69 (−1.63, 5.00)	0.315	2.74 (−0.47, 5.95)	0.094	0.763
Low-exposure group	38 (33.33)	Ref		Ref		
Medium-exposure group	38 (33.33)	1.55 (−4.16, 7.27)	0.590	0.23 (−5.54, 6.00)	0.937	
High-exposure group	38 (33.34)	5.24 (0.83, 9.65)	0.021	−1.61 (−7.45, 4.23)	0.583	
P-trend			0.061		0.540	
LnDIN		−3.21 (−5.35, −1.08)	0.004	−2.04 (−4.04, −0.03)	0.046	0.140
Low-exposure group	38 (33.33)	Ref		Ref		
Medium-exposure group	38 (33.33)	−4.74 (−9.64, 0.16)	0.058	−2.38 (−7.37, 2.62)	0.345	
High-exposure group	38 (33.34)	−9.47 (−15.03, −3.92)	0.001	−7.00 (−12.77, −1.23)	0.018	
P-trend			<0.001		0.011	
LnNACE		−2.12 (−4.22, −0.02)	0.048	0.15 (−2.00, 2.29)	0.893	0.763
Low-exposure group	38 (33.33)	Ref		Ref		
Medium-exposure group	38 (33.33)	0.89 (−4.58, 6.37)	0.746	2.14 (−3.01, 7.30)	0.409	
High-exposure group	38 (33.34)	−3.34 (−9.03, 2.35)	0.245	2.46 (−3.58, 8.50)	0.419	
P-trend			0.234		0.458	
LnCLO		−2.92 (−5.29, −0.55)	0.016	−3.24 (−5.37, −1.11)	0.003	0.060
Low-exposure group	38 (33.33)	Ref		Ref		
Medium-exposure group	38 (33.33)	0.08 (−4.68, 4.84)	0.974	0.64 (−3.72, 5.00)	0.771	
High-exposure group	38 (33.34)	−2.05 (−7.78, 3.67)	0.477	−1.38 (−6.95, 4.19)	0.622	
P-trend			0.465		0.345	
LnFLU		1.09 (−1.79, 3.97)	0.455	0.64 (−2.09, 3.37)	0.644	0.859
<LOD	79 (69.30)	Ref		Ref		
≥LOD	35 (30.70)	2.19 (−2.71, 7.10)	0.378	1.77 (−2.87, 6.40)	0.451	
LnSUL		−1.36 (−4.01, 1.28)	0.310	−1.62 (−3.99, 0.74)	0.177	0.763
<LOD	43 (37.72)	Ref		Ref		
LOD-Median	36 (31.58)	−0.02 (−5.26, 5.23)	0.994	−0.30 (−4.85, 4.25)	0.896	
>Median	35 (30.70)	0.12 (−5.08, 5.33)	0.962	−1.14 (−6.35, 4.08)	0.665	
P-trend			0.966		0.674	
LnTHIA		−2.82 (−8.00, 2.36)	0.283	−2.63 (−7.54, 2.27)	0.289	0.763
<LOD	96 (84.21)	Ref		Ref		
≥LOD	18 (15.79)	−1.74 (−7.97, 4.48)	0.580	0.16 (−5.67, 5.98)	0.958	
LnIMI		−1.41 (−3.10, 0.28)	0.100	−0.91 (−2.43, 0.61)	0.237	0.520
Low-exposure group	38 (33.33)	Ref		Ref		
Medium-exposure group	38 (33.33)	0.55 (−4.25, 5.35)	0.819	4.99 (0.12, 9.86)	0.055	
High-exposure group	38 (33.34)	−2.21 (−8.13, 3.70)	0.459	−1.30 (−6.57, 3.96)	0.622	
P-trend			0.432		0.544	
LnNIT		0.73 (−0.72, 2.18)	0.323	−0.02 (−1.42, 1.39)	0.983	0.877
Low-exposure group	38 (33.33)	Ref		Ref		
Medium-exposure group	38 (33.33)	−0.21 (−6.25, 5.83)	0.945	2.62（0.79，9.49）	0.124	
High-exposure group	38 (33.34)	2.42 (−3.68, 8.52)	0.431	−2.03 (−6.23, 2.17)	0.339	
P-trend			0.389	0.174		
LnTMX		−0.78 (−4.50, 2.93)	0.677	−0.50 (−3.85, 2.86)	0.770	0.911
<LOD	58 (50.88)	Ref		Ref		
≥LOD	56 (49.12)	−2.58 (−7.10, 1.94)	0.261	0.64 (0.24，1.67)	0.369	

Abbreviations: β, coefficient of regression; CI, Confidence Interval; *FDR*, false discovery rate; [a] unadjusted; [b] adjusted for prepregnancy BMI, maternal education, high-risk pregnancy, children's gender, children's age, gestational age, delivery way.

Table S3. Association between cord plasma NEOs levels and risk of low-average intelligence (*N* = 114).

NEOs (ng/mL)	*N* (%)	Model 1[a]		Model 2[b]		
		OR (95% CI)	*p* value	OR (95% CI)	*p* value	*FDR*
LnACE		0.66 (0.34, 1.16)	0.167	0.47 (0.21, 0.95)	0.043	0.438
Low-exposure group	38 (33.33)	Ref		Ref		
Medium-exposure group	38 (33.33)	0.48 (0.17, 1.26)	0.142	0.53 (0.11, 2.38)	0.412	
High-exposure group	38 (33.34)	0.53 (0.19, 1.43)	0.215	1.03 (0.20, 5.50)	0.971	
P-trend			0.226		0.516	
LnDIN		1.65 (1.11, 2.56)	0.018	1.66 (1.05, 2.76)	0.038	0.400
Low-exposure group	38 (33.33)	Ref		Ref		
Medium-exposure group	38 (33.33)	2.45 (0.97, 6.48)	0.063	3.01 (0.82, 12.23)	0.104	
High-exposure group	38 (33.34)	3.75 (1.41, 10.72)	0.010	4.41 (1.38, 15.66)	0.015	
P-trend			0.009		0.025	
LnNACE		1.07 (0.74, 1.56)	0.725	0.68 (0.40, 1.11)	0.134	0.438
Low-exposure group	38 (33.33)	Ref		Ref		
Medium-exposure group	38 (33.33)	0.71 (0.27, 1.81)	0.473	0.30 (0.07, 1.08)	0.080	
High-exposure group	38 (33.34)	1.13 (0.42, 3.05)	0.803	0.24 (0.04, 1.23)	0.108	
P-trend			0.808		0.146	
LnCLO		1.13 (0.75, 1.78)	0.566	1.16 (0.71, 1.98)	0.579	0.626
Low-exposure group	38 (33.33)	Ref		Ref		
Medium-exposure group	38 (33.33)	1.13 (0.43, 2.96)	0.807	1.25 (0.32, 5.04)	0.749	
High-exposure group	38 (33.34)	1.00 (0.39, 2.60)	1.000	0.72 (0.20, 2.52)	0.615	
P-trend			1.000		0.795	
LnFLU		0.84 (0.52, 1.38)	0.482	0.86 (0.48, 1.57)	0.614	0.791
<LOD	79 (69.30)	Ref		Ref		
≥LOD	35 (30.70)	0.78 (0.34, 1.83)	0.566	0.89 (0.32, 2.54)	0.820	
LnSUL		1.25 (0.79, 2.05)	0.352	1.30 (0.75, 2.34)	0.360	0.621
<LOD	43 (37.72)	Ref		Ref		
LOD-Median	36 (31.58)	1.35 (0.53, 3.51)	0.535	2.46 (0.68, 10.23)	0.187	
>Median	35 (30.70)	1.29 (0.51, 3.38)	0.594	1.51 (0.49, 4.85)	0.473	
P-trend			0.574		0.542	
LnTHIA		1.02 (0.43, 2.75)	0.962	0.71 (0.24, 2.10)	0.525	0.732
<LOD	96 (84.21)	Ref		Ref		
≥LOD	18 (15.79)	0.75 (0.27, 2.21)	0.587	0.37 (0.10, 1.32)	0.125	
LnIMI		1.21 (0.89, 1.66)	0.231	1.19 (0.85, 1.70)	0.326	0.438
Low-exposure group	38 (33.33)	Ref		Ref		
Medium-exposure group	38 (33.33)	0.89 (0.35, 2.29)	0.811	0.27 (0.06, 1.04)	0.069	
High-exposure group	38 (33.34)	1.28 (0.48, 3.41)	0.622	1.25 (0.34, 4.76)	0.734	
P-trend			0.627		0.693	
LnNIT		1.01 (0.79, 1.30)	0.948	1.23 (0.90, 1.71)	0.196	0.438
Low-exposure group	38 (33.33)	Ref		Ref		
Medium-exposure group	38 (33.33)	1.43 (0.55, 3.81)	0.465	2.16 (0.65, 7.89)	0.220	
High-exposure group	38 (33.34)	1.12 (0.44, 2.90)	0.811	1.87 (0.46, 8.40)	0.389	
P-trend			0.808		0.232	
LnTMX		0.70 (0.36, 1.32)	0.272	0.62 (0.27, 1.26)	0.199	0.438
<LOD	58 (50.88)	Ref		Ref		
≥LOD	56 (49.12)	0.95 (0.43, 2.07)	0.895	0.73 (0.28, 1.82)	0.496	

Abbreviations: OR, Odds Ratio, CI, Confidence Interval; *FDR*, false discovery rate; [a] unadjusted; [b] adjusted for prepregnancy BMI, maternal education, high-risk pregnancy, children's gender, children's age, gestational age, delivery way.

Table S4. Correlation between cord plasma NEOs concentration (ng/mL) and ASQ score (N = 114) (unadjusted).

NEOs (ng/mL)	Communication		Gross motor		Fine motor		Problem solving		Individual-society	
	**β (95% CI)**	***p* value**	**β (95% CI)**	***p* value**	**β (95% CI)**	***p* value**	**β (95% CI)**	***p* value**	**β (95% CI)**	***p* value**
LnACE	0.00 (−0.03, 0.04)	0.798	−0.00 (−0.04, 0.04)	0.987	0.00 (−0.04, 0.04)	0.836	0.02 (−0.02, 0.05)	0.395	−0.01 (−0.05, 0.02)	0.506
LnDIN	0.01 (−0.01, 0.04)	0.279	0.00 (−0.02, 0.03)	0.876	**0.03 (0.00, 0.05)**	**0.042**	−0.00 (−0.02, 0.02)	0.995	0.00 (−0.02, 0.03)	0.911
LnNACE	0.01 (−0.01, 0.03)	0.417	0.01 (−0.01, 0.04)	0.288	0.00 (−0.02, 0.03)	0.892	0.00 (−0.02, 0.03)	0.778	0.01 (−0.01, 0.03)	0.339
LnCLO	0.00 (−0.02, 0.03)	0.861	−0.01 (−0.03, 0.02)	0.687	**0.04 (0.01, 0.07)**	**0.004**	−0.01 (−0.03, 0.02)	0.652	−0.01 (−0.03, 0.02)	0.617
LnFLU	−0.01 (−0.04, 0.02)	0.653	−0.02 (−0.05, 0.02)	0.317	**−0.04 (−0.07, −0.00)**	**0.035**	0.01 (−0.03, 0.04)	0.660	−0.01 (−0.04, 0.02)	0.457
LnSUL	0.01 (−0.02, 0.04)	0.670	0.00 (−0.03, 0.03)	0.857	0.02 (−0.01, 0.06)	0.127	0.01 (−0.02, 0.04)	0.424	0.01 (−0.02, 0.04)	0.457
LnTHIA	−0.06 (−0.12, 0.00)	0.060	−0.03 (−0.09, 0.03)	0.316	0.00 (−0.06, 0.06)	0.946	−0.04 (−0.10, 0.02)	0.229	−0.01 (−0.07, 0.05)	0.711
LnIMI	−0.01 (−0.03, 0.00)	0.137	−0.02 (−0.03, 0.00)	0.112	−0.00 (−0.02, 0.02)	0.701	0.00 (−0.02, 0.02)	0.728	−0.00 (−0.02, 0.02)	0.967
LnNIT	0.00 (−0.01, 0.02)	0.806	0.01 (−0.01, 0.03)	0.237	0.01 (−0.01, 0.02)	0.503	0.00 (−0.02, 0.02)	0.910	−0.00 (−0.02, 0.02)	0.987
LnTMX	0.02 (−0.02, 0.06)	0.252	−0.04 (−0.08, 0.01)	0.100	−0.04 (−0.09, 0.00)	0.070	−0.00 (−0.04, 0.04)	0.987	−0.01 (−0.05, 0.03)	0.673

Abbreviations: β, standardized regression coefficient; CI, Confidence Interval.

Table S5. Correlation between cord plasma NEOs concentration (ng/mL) and ASQ score (N = 114) (adjusted).

NEOs (ng/ml)	Communication			Gross motor			Fine motor			Problem solving			Individual-society		
	β (95% CI)	*p* value	*FDR*	β (95% CI)	*p* value	*FDR*	β (95% CI)	*p* value	*FDR*	β (95% CI)	*p* value	*FDR*	β (95% CI)	*p* value	*FDR*
LnACE	0.01 (−0.03, 0.05)	0.515	0.706	0.01 (−0.03, 0.05)	0.658	0.658	0.03 (−0.01, 0.07)	0.184	0.307	0.03 (−0.01, 0.07)	0.160	0.800	−0.00 (−0.04, 0.04)	0.962	0.995
LnDIN	0.02 (−0.01, 0.05)	0.124	0.420	−0.01 (−0.03, 0.02)	0.492	0.658	0.02 (−0.01, 0.05)	0.144	0.288	−0.00 (−0.03, 0.03)	0.989	0.989	−0.00 (−0.03, 0.02)	0.815	0.995
LnNACE	0.02 (−0.01, 0.04)	0.242	0.605	0.01 (−0.02, 0.03)	0.571	0.658	−0.01 (−0.04, 0.02)	0.589	0.616	0.01 (−0.02, 0.03)	0.551	0.918	0.01 (−0.02, 0.03)	0.482	0.995
LnCLO	−0.00 (−0.03, 0.03)	0.920	0.920	−0.01 (−0.04, 0.02)	0.450	0.658	0.03 (−0.00, 0.06)	0.061	0.203	−0.01 (−0.04, 0.02)	0.451	0.918	−0.01 (−0.04, 0.02)	0.423	0.995
LnFLU	−0.01 (−0.04, 0.02)	0.565	0.706	−0.01 (−0.05, 0.02)	0.407	0.658	−0.03 (−0.07, 0.01)	0.118	0.288	0.00 (−0.03, 0.03)	0.946	0.989	−0.01 (−0.04, 0.03)	0.659	0.995
LnSUL	0.01 (−0.02, 0.04)	0.547	0.706	0.01 (−0.02, 0.04)	0.635	0.658	0.03 (−0.00, 0.06)	0.057	0.203	0.02 (−0.01, 0.05)	0.278	0.918	0.01 (−0.02, 0.04)	0.529	0.995
LnTHIA	−0.07 (−0.13, −0.01)	0.030	0.300	−0.04 (−0.11, 0.02)	0.164	0.410	−0.02 (−0.09, 0.05)	0.589	0.616	−0.06 (−0.12, 0.00)	0.065	0.650	−0.02 (−0.08, 0.04)	0.447	0.995
LnIMI	−0.02 (−0.03, 0.00)	0.126	0.420	−0.02 (−0.04, −0.00)	0.038	0.190	−0.01 (−0.03, 0.01)	0.512	0.616	−0.00 (−0.02, 0.02)	0.937	0.989	−0.00 (−0.02, 0.02)	0.980	0.995
LnNIT	−0.00 (−0.02, 0.02)	0.902	0.920	0.01 (−0.00, 0.03)	0.095	0.317	0.00 (−0.01, 0.02)	0.616	0.616	0.00 (−0.01, 0.02)	0.785	0.989	−0.00 (−0.02, 0.02)	0.995	0.995
LnTMX	0.02 (−0.02, 0.06)	0.383	0.706	−0.05 (−0.09, −0.01)	0.024	0.190	−0.06 (−0.11, −0.01)	0.011	0.110	−0.02 (−0.06, 0.03)	0.463	0.918	−0.02 (−0.06, 0.02)	0.421	0.995

Abbreviations: β, standardized regression coefficient; CI, Confidence Interval; *FDR*, false discovery rate; Adjusted for prepregnancy BMI, maternal education, high-risk pregnancy, children's gender, children's age, gestational age, delivery way.

Table S6. Stratified analysis of 10 cord plasma NEOs and ASQ scores.

NEOs (ng/mL)	Communication		Gross motor		Fine motor		Problem solving		Individual-society	
	β (95% CI)	*p* value	β (95% CI)	*p* value	β (95% CI)	*p* value	β (95% CI)	*p* value	β (95% CI)	*p* value
LnACE										
Boys	0.67 (−2.24, 3.59)	0.645	0.43 (−2.69, 3.55)	0.784	1.41 (−2.69, 5.52)	0.492	1.53 (−1.10, 4.15)	0.248	−1.30 (−4.29, 1.68)	0.384
Girls	0.18 (−2.5, 2.87)	0.891	−1.38 (−4.64, 1.87)	0.398	0.97 (−4.92, 6.86)	0.742	1.23 (−1.97, 4.43)	0.442	0.34 (−1.99, 2.67)	0.771
LnDIN										
Boys	1.42 (−0.85, 3.69)	0.216	0.78 (−1.68, 3.24)	0.528	2.74 (−0.42, 5.91)	0.088	−0.13 (−2.23, 1.97)	0.902	−0.69 (−3.07, 1.68)	0.559
Girls	0.82 (−0.67, 2.31)	0.273	−1.44 (−3.24, 0.35)	0.112	−0.66 (−3.96, 2.64)	0.691	−0.28 (−2.09, 1.52)	0.753	0.59 (−0.71, 1.88)	0.367
LnNACE										
Boys	0.38 (−1.65, 2.41)	0.708	0.80 (−1.36, 2.96)	0.461	−1.26 (−4.11, 1.59)	0.380	0.36 (−1.49, 2.21)	0.700	0.20 (−1.90, 2.29)	0.851
Girls	0.81 (−0.88, 2.50)	0.340	0.58 (−1.5, 2.65)	0.580	0.14 (−3.61, 3.88)	0.942	0.67 (−1.36, 2.71)	0.509	0.89 (−0.57, 2.35)	0.228
LnCLO										
Boys	0.01 (−2.7, 2.71)	0.995	−0.40 (−3.29, 2.49)	0.781	−0.20 (−4.02, 3.62)	0.916	−1.81 (−4.22, 0.59)	0.136	−0.97 (−3.75, 1.80)	0.484
Girls	−0.17 (−1.78, 1.43)	0.830	0.30 (−1.66, 2.26)	0.760	2.12 (−1.35, 5.60)	0.226	0.16 (−1.77, 2.08)	0.870	0.37 (−1.02, 1.76)	0.599
LnFLU										
Boys	−0.72 (−3.56, 2.12)	0.614	−0.65 (−3.69, 2.39)	0.670	−2.4 (−6.36, 1.57)	0.230	0.03 (−2.57, 2.63)	0.981	−0.61 (−3.54, 2.33)	0.680
Girls	−0.17 (−2.08, 1.75)	0.860	−0.61 (−2.94, 1.72)	0.601	−0.14 (−4.34, 4.06)	0.945	0.72 (−1.56, 3.00)	0.531	0.08 (−1.58, 1.74)	0.920
LnSUL										
Boys	0.16 (−2.44, 2.77)	0.900	1.29 (−1.46, 4.05)	0.351	−0.14 (−3.81, 3.54)	0.941	0.97 (−1.39, 3.32)	0.414	1.34 (−1.31, 3.99)	0.315
Girls	0.37 (−1.40, 2.14)	0.673	−0.16 (−2.32, 2.01)	0.883	2.1 (−1.75, 5.94)	0.278	0.47 (−1.64, 2.59)	0.655	0.14 (−1.40, 1.68)	0.858
LnTHIA										
Boys	−4.39 (−9.45, 0.68)	0.088	−4.25 (−9.69, 1.19)	0.123	−6.3 (−13.44, 0.85)	0.083	−7.66 (−11.86, −3.45)	**0.001**	−3.42 (−8.71, 1.87)	0.200
Girls	−3.18 (−6.65, 0.3)	0.073	0.31 (−4.08, 4.7)	0.887	4.13 (−3.67, 11.93)	0.292	2.04 (−2.23, 6.30)	0.342	1.05 (−2.05, 4.15)	0.500
LnIMI										

NEOs (ng/mL)	Communication		Gross motor		Fine motor		Problem solving		Individual-society	
	**β (95% CI)**	***p* value**	**β (95% CI)**	***p* value**	**β (95% CI)**	***p* value**	**β (95% CI)**	***p* value**	**β (95% CI)**	***p* value**
Boys	0.18 (−1.82, 2.19)	0.855	−1.25 (−3.37, 0.86)	0.239	−2.35 (−5.10, 0.40)	0.092	−1.44 (−3.21, 0.34)	0.111	−0.81 (−2.86, 1.25)	0.435
Girls	−1.05 (−2.09, −0.02)	**0.047**	−1.17 (−2.44, 0.11)	0.072	0.84 (−1.51, 3.19)	0.475	1.26 (0.02, 2.50)	**0.046**	0.49 (−0.44, 1.41)	0.295
LnNIT										
Boys	−0.08 (−1.49, 1.33)	0.914	1.13 (−0.34, 2.60)	0.128	0.65 (−1.33, 2.63)	0.513	0.87 (−0.39, 2.13)	0.172	0.41 (−1.04, 1.86)	0.575
Girls	−0.41 (−1.41, 0.58)	0.408	0.05 (−1.18, 1.27)	0.940	−0.59 (−2.79, 1.6)	0.590	−0.76 (−1.94, 0.43)	0.205	−0.66 (−1.51, 0.19)	0.124
LnTMX										
Boys	0.44 (−3.69, 4.58)	0.830	−5.79 (−9.88, −1.70)	**0.007**	−5.61 (−11.22, 0.01)	0.050	−0.21 (−3.98, 3.55)	0.909	−2.49 (−6.70, 1.71)	0.239
Girls	1.17 (−1.09, 3.42)	0.304	−0.42 (−3.20, 2.36)	0.764	−1.32 (−6.30, 3.67)	0.598	−1.67 (−4.35, 1.02)	0.218	0.57 (−1.40, 2.54)	0.561

Abbreviations: β, standardized regression coefficient; CI, Confidence Interval.

Table S7. Interaction analysis of 10 cord plasma NEOs and ASQ scores.

NEOs (ng/mL)	Communication	Gross movement	Fine movement	Problem solving	Individual-society
	***p* for interaction**	***p* for interaction**	***p* for interaction**	***p* for interaction**	***p* for interaction**
LnACE	0.999	0.285	0.977	0.892	0.534
LnDIN	0.853	0.246	0.145	0.785	0.311
LnNACE	0.940	0.410	0.620	0.885	0.999
LnCLO	0.999	0.354	0.318	0.162	0.198
LnFLU	0.756	0.975	0.392	0.578	0.715
LnSUL	0.978	0.426	0.466	0.437	0.504
LnTHIA	0.913	0.162	0.111	0.008	0.147
LnIMI	0.315	0.780	0.073	0.006	0.377
LnNIT	0.761	0.323	0.468	0.074	0.293
LnTMX	0.949	0.023	0.331	0.590	0.174
